# Cx43 can form functional channels at the nuclear envelope and modulate gene expression in cardiac cells

**DOI:** 10.1098/rsob.230258

**Published:** 2023-11-01

**Authors:** Tania Martins-Marques, Katja Witschas, Ilda Ribeiro, Mónica Zuzarte, Steve Catarino, Teresa Ribeiro-Rodrigues, Francisco Caramelo, Trond Aasen, Isabel Marques Carreira, Lino Goncalves, Luc Leybaert, Henrique Girao

**Affiliations:** ^1^ Univ Coimbra, Coimbra Institute for Clinical and Biomedical Research (iCBR), Faculty of Medicine, 3000-548 Coimbra, Portugal; ^2^ Univ Coimbra, Center for Innovative Biomedicine and Biotechnology (CIBB), 3004-504 Coimbra, Portugal; ^3^ Clinical Academic Centre of Coimbra (CACC), 3004-561 Coimbra, Portugal; ^4^ Department of Basic Medical Sciences – Physiology group, Ghent University, 9000 Ghent, Belgium; ^5^ Univ Coimbra, Cytogenetics and Genomics Laboratory (CIMAGO), Faculty of Medicine, 3004-531 Coimbra, Portugal; ^6^ Univ Coimbra, Laboratory of Biostatistics and Medical Informatics, Faculty of Medicine, 3004-531 Coimbra, Portugal; ^7^ Patologia Molecular Translacional, Vall d'Hebron Institut de Recerca (VHIR), Vall d'Hebron Hospital Universitari, Vall d'Hebron Barcelona Hospital Campus, Passeig Vall d'Hebron 119-129, 08035 Barcelona, Spain; ^8^ CIBER de Cáncer (CIBERONC), Instituto de Salud Carlos III, Avenida de Monforte de Lemos 3-5, 28029 Madrid, Spain

**Keywords:** connexin43, nuclear translocation, subcellular trafficking, gene expression

## Abstract

Classically associated with gap junction-mediated intercellular communication, connexin43 (Cx43) is increasingly recognized to possess non-canonical biological functions, including gene expression regulation. However, the mechanisms governing the localization and role played by Cx43 in the nucleus, namely in transcription modulation, remain unknown. Using comprehensive and complementary approaches encompassing biochemical assays, super-resolution and immunogold transmission electron microscopy, we demonstrate that Cx43 localizes to the nuclear envelope of different cell types and in cardiac tissue. We show that translocation of Cx43 to the nucleus relies on Importin-β, and that Cx43 significantly impacts the cellular transcriptome, likely by interacting with transcriptional regulators. *In vitro* patch-clamp recordings from HEK293 and adult primary cardiomyocytes demonstrate that Cx43 forms active channels at the nuclear envelope, providing evidence that Cx43 can participate in nucleocytoplasmic shuttling of small molecules. The accumulation of nuclear Cx43 during myogenic differentiation of cardiomyoblasts is suggested to modulate expression of genes implicated in this process. Altogether, our study provides new evidence for further defining the biological roles of nuclear Cx43, namely in cardiac pathophysiology.

## Graphical abstract

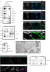

## 1. Introduction

The nucleus encloses the genetic patrimony of eukaryotic cells, enabling the compartmentalization of gene transcription, pre-mRNA splicing and ribosome assembly [1]. The structural integrity of the nucleus is primarily maintained by the nuclear envelope, a double phospholipid bilayer comprising the outer nuclear membrane (ONM) and the inner nuclear membrane (INM) that merge where the nuclear pore complexes (NPCs) are embedded [2]. Although the ONM is continuous with the peripheral endoplasmic reticulum (ER), some proteins specifically localize within the ONM, such as proteins of the linker of nucleoskeleton and cytoskeleton (LINC) complex that include Nesprins [2,3]. The INM is also composed of a unique subset of integral membrane proteins that provide anchorage to the nuclear lamina and binding to chromatin organization regulators [4]. Not surprisingly, disruption of nuclear envelope integrity due to mutations or loss of nuclear envelope components, such as Lamins, Nesprins or INM proteins, has been associated with several human diseases, ranging from muscular dystrophies to progeria, collectively named laminopathies [5,6]. Nonetheless, the trafficking routes and regulatory mechanisms underlying the transport of proteins, including membrane proteins, to the nuclear envelope are poorly established.

Canonically associated with gap junction (GJ)-mediated communication (GJIC), Connexin43 (Cx43) ensures electric and metabolic coupling between adjacent cells, which is particularly important in the heart [7,8]. Recent compelling evidence implicates Cx43 in other biological functions, including cell adhesion, gene expression regulation and mitochondrial bioenergetics [9,10]. Previous proteomic studies have identified multiple nuclear proteins as Cx43 interactors, supporting the emerging revolutionizing concept of non-canonical Cx43 functions, namely at the nuclear level [9,11]. Several human pathologies, including cardiac disorders, have been associated with remodelling of Cx43 that mainly involves changes in Cx43 expression and localization [5]. Up until recently, Cx43 remodelling implicated in cardiac diseases was limited to Cx43 downregulation, internalization and lateralization leading to GJIC derailment. However, recent studies have associated Cx43 remodelling with other processes and phenotypes. Accordingly, we demonstrated that heart ischemia induces changes in the secretion of Cx43 in extracellular vesicles [12].

Although total Cx43 levels and cell surface-localized hemichannel activity have been associated with alterations in gene transcription, the relevance of the nuclear localization of full-length Cx43 to these mechanisms has not been unraveled so far [13,14]. By contrast with the full-length protein, the impact of nuclei-localized truncated Cx43 isoforms upon transcription of apoptosis-related genes and N-cadherin was previously demonstrated [15–17]. Importantly, several DNA- and RNA-binding motifs were identified on the Cx43 sequence, supporting a direct role for Cx43 in gene expression regulation [18,19]. Besides physical interaction with nuclear components, it is plausible that the function of nuclear Cx43 relies on its channel activity. However, this has never been demonstrated.

Although the activation of protein kinase A (PKA) and Wnt signalling have been implicated in the accumulation of Cx43 in the nucleus, the trafficking routes whereby Cx43 is targeted to the nucleus, and its biological relevance and function remain largely unknown [20,21]. Some valuable hints may be given by studies on other cell surface receptors, including ErbB-2 and epidermal growth factor receptor (EGFR), which can also be found at the INM [22]. Nuclear transport of plasma membrane-localized EGFR follows ligand-induced internalization, involving retrograde Golgi-to-ER or integral trafficking from the ER to the nuclear envelope transport (Internet) pathways, whereas ErbB-2 relies on the recognition of nuclear localization signals (NLS) by Importin-β after clathrin-mediated endocytosis [22,23].

In this study, we gather compelling evidence that Cx43 localizes at the nuclear envelope of a wide variety of cell types and interacts with well-known nuclear proteins, namely Emerin, Nesprin3, Lamin A/C and Lamin B. Proteomic analyses uncovered novel interacting partners of nuclear Cx43, including transcription factors and regulators of nucleocytoplasmic trafficking. In addition, our data provide valuable mechanistic insights, showing that transport of Cx43 to the nucleus is mediated by Importin-β. Moreover, we demonstrate that Cx43 forms functional channels at the nuclear envelope, also in cardiomyocytes, and that Cx43 impacts the gene expression landscape of cultured cells. We further show that myogenic differentiation of H9c2 cardiomyoblasts is associated with an accumulation of Cx43 in the nucleus, and by changes in genes whose expression is regulated by Cx43. Overall, our study delineates new avenues for further defining the biological roles of nuclear Cx43, particularly in cardiac pathophysiology.

## 2. Results

### 2.1. Cx43 is localized at the nuclear envelope of cultured cells

**Figure 1.**
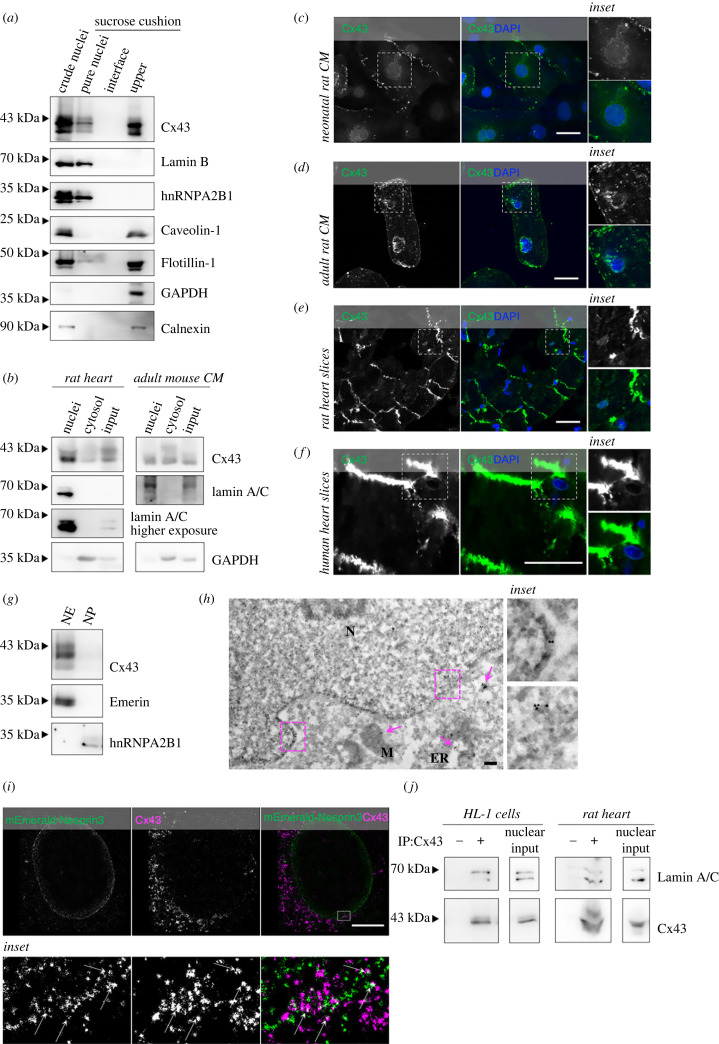
Cx43 is localized at the nuclear envelope of cultured cells and cardiac tissue. (*a*) Crude nuclei isolated from HEK293^Cx43+^ cells were resuspended in 1% TX100, layered onto a 1.8 M sucrose cushion and subjected to high-speed centrifugation. Pellet (purified nuclei), interface and upper fractions were collected and analysed by WB. Lamin B and hnRNPA2B1 served as nuclear markers, caveolin-1 and flotillin-1 as markers of lipid rafts, GAPDH as cytosolic marker, and Calnexin was used as an ER marker. (*b*) Nuclei isolated from heart lysates or adult rat cardiomyocytes (CM) were resuspended in 1% TX100, layered onto a 1.8 M sucrose cushion and subjected to high-speed centrifugation. Purified nuclei, cytosolic fractions and inputs were analysed by WB. (*c–f*) Representative confocal microscopy images of neonatal rat cardiomyocytes (*c*), adult rat cardiomyocytes (*d*), rat heart slices (*e*) and human heart slices (*f*) stained for Cx43 (green; antibody AB0016, SICGEN). Nuclei were stained with DAPI. Inset images display a magnified view of the boxed region. Scale bars, 20 µm. (*g*) Nuclear envelopes were prepared from purified nuclei of HEK293^Cx43+^ cells and analysed by WB. Emerin was used as a nuclear envelope (NE) marker and hnRNPA2B1 as a nucleoplasm (NP) marker. (*h*) Representative TEM-immunogold image of HEK293^Cx43+^ cells stained for Cx43 (antibody 710700, Thermo Fisher Scientific). Arrows highlight positive Cx43 labelling in cytosolic vesicles, ER and mitochondria. N: nucleus; M: mitochondria; ER: endoplasmic reticulum. Scale bars, 200 nm. (*i*) Single-molecule localization microscopy analysis of nuclear Cx43 in cardiac mouse endothelial MCEC cells transfected with mEmerald-Nesprin3 for 24 h. Live cells were treated with 3% TX100, before immunostaining with antibodies against Cx43 (magenta; antibody AB0016, SICGEN) and GFP (green). Inset images display a magnified view of the boxed region. Arrows highlight co-localization points. Scale bars, 5 µm. (*j*) Cx43 was immunoprecipitated (IP) from purified nuclear extracts of HL-1 murine cardiomyocytes or rat heart lysates, as indicated, after which interaction with Lamin A/C was assessed by WB.

Although the presence of Cx43 in the nucleus has been shown, the precise localization, function and biological relevance of nuclear Cx43 remain largely unknown [9,17]. Since previous subcellular fractionation approaches to disclose the presence of Cx43 in the nucleus resorted to techniques that cannot discard contamination with other organelles, in this study, we started by optimizing meticulous biochemical assays that yield more pure nuclear extracts to accurately unveil the dynamics and homeostasis of Cx43 localized to the nucleus. We started by optimizing our experimental approach in HEK293 cells that are more amenable for genetic manipulation. Given that parental cells express very low levels of Cx43, we generated a cell line with stable expression of Cx43 of rat origin (HEK293^Cx43+^; electronic supplementary material, figure S1*a*). The generated monoclonal cell line was selected based on a low level of Cx43 overexpression and a subcellular distribution similar to that found in cell lines with endogenous Cx43. Our results (electronic supplementary material, figure S1*b*) show that full-length Cx43 is present in crude nuclei extracts, where it is resistant to 7 M urea extraction, but can be solubilized by 1% TX100 and increasing salt concentrations, suggesting that Cx43 behaves as an integral membrane protein of the nucleus [24]. As a control, a soluble nuclear protein (hnRNPA2B1) was completely extracted by 7 M urea. However, the presence of Caveolin-1 in nuclear extracts, with a partition profile similar to that of Cx43, pointed to contamination with lipid rafts [25]. Therefore, we proceed to the establishment of a purification protocol of nuclear-enriched pellets, by adding 1% TX100 to crude nuclei, to solubilize the ONM/ER, followed by a sucrose cushion ultracentrifugation (electronic supplementary material, figure S1*c*). This was revealed to be a suitable strategy to considerably reduce lipid raft and ER contamination, with Caveolin-1, Flotillin-1 and Calnexin remaining at the upper cushion fraction (figure 1*a*). More importantly, full-length Cx43 was still detected in the pure nuclei fraction (figure 1*a*), together with an enrichment in the nuclear proteins Lamin B and hnRNPA2B1. A strong signal of Cx43 was also observed in the upper cushion fraction, likely representing GJ and ER/ONM-associated Cx43.

To discard possible artefacts induced by overexpression of Cx43 in HEK293 cells, we employed and validated our nuclei purification protocol in multiple cell lines with endogenous expression of Cx43, which demonstrated that full-length Cx43 is also present in purified nuclei of cardiomyoblast rat H9c2, cancer human C33a and renal epithelial rat NRK cells (electronic supplementary material, figure S1*d*). The localization of Cx43 in the nucleus of H9c2 cells suggests that Cx43 can also be found at the nucleus of cells forming cardiac tissues, where Cx43 is abundantly expressed. Data depicted in figure 1*b* clearly demonstrate that Cx43 localizes in nuclei purified from total rat heart extracts and isolated primary adult mouse cardiomyocytes, excluding possible artefacts induced by cell culture. These results were corroborated by confocal microscopy analysis in cultured primary cardiomyocytes from neonatal (figure 1*c*) and adult (figure 1*d*) rats, as well as *in vivo*, in slices from rat hearts (figure 1*e*), in which the staining of Cx43 in the nucleus was noticeable, besides the classical localization at the plasma membrane. We also obtained myocardial biopsies from human hearts of patients with aortic stenosis, in which despite the pathological remodelling of cell surface Cx43 channels, we also observed staining of Cx43 in the nucleus (figure 1*f*).

Next, we sought to investigate the subnuclear localization of Cx43, following fractionation of nuclear pellets into nuclear envelope and nucleoplasm fractions. As expected for a transmembrane protein, Cx43 was predominantly found at the nuclear envelope fraction (figure 1*g*), which was further supported by the sensitivity of nuclear Cx43 to trypsin-mediated cleavage (electronic supplementary material, figure S1*e*). Importantly, nuclear Cx43 is phosphorylated, similar to what is observed at the plasma membrane (electronic supplementary material, figure S1*f*).

Consistent with a localization of Cx43 at the nuclear envelope, Cx43 staining at the perinuclear and nuclear region was resistant to detergent extraction after incubation of live HEK293^Cx43+^ and H9c2 cells with buffer containing 3% TX100, which also preserved larger GJ plaques, classically described as TX100-insoluble structures (electronic supplementary material, figure S1*g–h*, bottom panels). TX100 extraction removed most cytosolic Cx43 labelling that is usually observed after standard permeabilization and immunofluorescence staining conditions, and likely increased the avidity of the antibody to more exposed Cx43 epitopes (electronic supplementary material, figure S1*g–h*, upper panels).

The localization of Cx43 to the nucleus was confirmed by transmission electron microscopy (TEM) that revealed Cx43-positive immunogold labelling at the nuclear envelope (figure 1*h*). Importantly, TEM-immunogold also uncovered Cx43 labelling in cytosolic vesicles, ER and mitochondria (figure 1*h*, arrows), consistent with its localization in other intracellular membranes, as previously demonstrated [26,27]. A complementary approach, resorting to the enhanced ascorbate peroxidase 2 (APEX2) genetic tag fused to Cx43 (Cx43-APEX2), was also employed. Our results (electronic supplementary material, figure S1*i*) show a positive TEM contrast of Cx43-APEX2 at the nuclear membrane (left panel), in addition to the localization of Cx43-APEX2 at the plasma membrane, forming GJ (right panel).

Analogous to what is observed at the plasma membrane, we hypothesized that nuclear Cx43 assembles into hexameric channels. Hence, to unveil the oligomerization state of Cx43, we analysed the electrophoretic migration profile of nuclear Cx43 in non-reducing conditions, which showed the presence of high molecular weight bands of Cx43 (approx. 80 and approx. 250 kDa), consistent with Cx43 dimers and hexamers (electronic supplementary material, figure S1*j*). This profile was observed in both purified and crude nuclei, supporting the idea that Cx43 assembles into hemichannels embedded both in the ONM and INM.

To validate the localization of Cx43 to the nucleus, we evaluated the association of Cx43 with well-known nuclear proteins. Confocal microscopy data show that Cx43 co-localizes with the INM protein Emerin and the ONM protein Nesprin3 (electronic supplementary material, figure S2*a–b*), which was further corroborated by super-resolution microscopy that revealed a partial co-localization of endogenous Cx43 particles with Nesprin3 (figure 1*i*). Moreover, co-immunoprecipitation (co-IP) in pure nuclear extracts show that Cx43 interacts with Nesprin3, Emerin, and with the nuclear intermediate filament protein Lamin B in HEK293 cells (electronic supplementary material, figure S2*c–e*), as well as with Lamin A/C in nuclei purified from cardiac cells and heart lysates (figure 1*j*). Interestingly, we preferentially detected interaction of Cx43 with Lamin C in nuclei from rat hearts, whereas in mouse HL-1 cells, nuclear Cx43 was found to interact mostly with Lamin A (figure 1*j*), suggesting that binding of Cx43 to either Lamin A or Lamin C can be cell type-specific.

Overall, resorting to different Cx43 antibodies and methodological approaches, our results provide clear evidence that Cx43 localizes to the nucleus of various cell types, including primary cardiac cells expressing endogenous Cx43, and cardiac tissue.

### 2.2. Proteomic analyses identify novel interacting partners of nuclear Cx43

**Figure 2.**
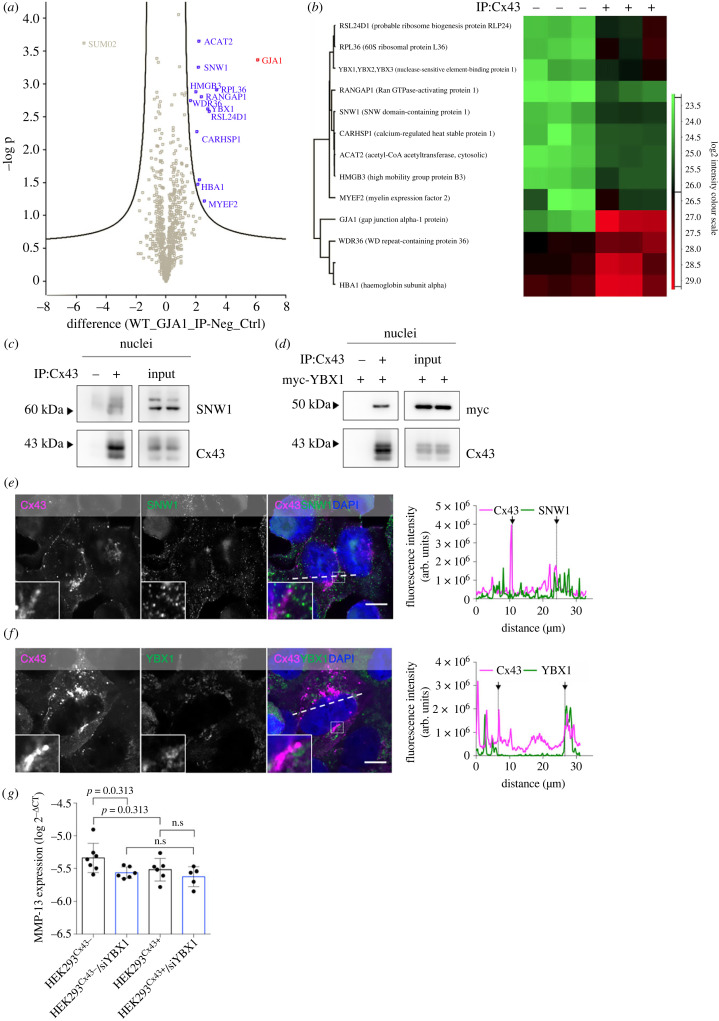
Cx43 interacts with several nuclear proteins. (*a*) 782 putative Cx43 interactors were quantified and subjected to statistical analysis (t-test), with a false discovery rate (FDR) = 0.01 and S_0_ = 1. Interaction levels between IP Cx43 and negative control samples were compared. (*b*) Heatmap representation of the 13 proteins selected as valid Cx43 interactors. (*c*) Cx43 was IP from purified nuclear extracts derived from HEK293^Cx43+^ followed by WB analysis using antibodies against SNW1. (*d*) Cx43 was IP from purified nuclear extracts of HEK293^Cx43+^ cells transfected with myc-YBX1 for 24 h. Interaction between Cx43 and myc-YBX1 was assessed by WB. (*e*,*f*). Co-localization between Cx43 and endogenous SNW1 (*e*) and YBX1 (*f*) was analysed by confocal microscopy. Line profile plots depict the intensity distribution of green and magenta channels along the dashed white lines in the merged image. Arrows in co-localization plots indicate the margins of the nucleus. Inset images display a magnified view of the boxed region. Scale bars, 10 µm. (*g*) Expression of *MMP-13* was analysed by real-time quantitative PCR (RT-qPCR) in HEK293^Cx43+^ and HEK293^Cx43−^, following siRNA-mediated knockdown of YBX1 for 48 h (siYBX1), where indicated. Results were normalized using r18s levels. Values are expressed as log2^−ΔCT^ (*n* = 5–7 biological replicates). n.s: non-significant.

Having established that Cx43 localizes to the nucleus, we next aimed to identify specific nuclear Cx43-binding partners that could contribute to regulate the levels and/or function of nuclear Cx43. To address that, we performed a proteomic analysis following IP of Cx43 from purified nuclear extracts. Our mass spectrometry (MS) data identified 13 Cx43 interactors, including transcription factors and chromatin-associated proteins (figure 2*a*,*b*; electronic supplementary material, table S1), in agreement with previous reports suggesting a role for Cx43 in regulating gene expression [16]. Gene ontology (GO)-term enrichment analysis linked nuclear Cx43 binding partners to mRNA stabilization and metabolism functions (electronic supplementary material, figure S3*a–d*).

Based on their biological relevance as transcriptional regulators, including in cardiac cells [28,29], we proceeded to validate the association between Cx43 and SNW domain-containing protein 1 (SNW1) and Y-box-binding protein 1 (YBX1). Our results demonstrate that Cx43 interacts with endogenous SNW1, YBX1, and overexpressed myc-YBX1 in pure nuclei (figure 2*c*,*d*; electronic supplementary material, figure S3*e*). Moreover, a partial co-localization between Cx43 and SNW1, YBX1 and myc-YBX1 was also observed, demonstrating the reliability of our MS approach (figure 2*e*,*f*; electronic supplementary material, figure S3*f*).

These data lead us to hypothesize that interaction between Cx43 and proteins with transcription factor activity, such as YBX1, can interfere with their DNA-binding functions, ultimately affecting gene expression. Therefore, we sought to assess the impact of Cx43 on YBX1-mediated gene transcription, comparing cells with stable expression of Cx43 (HEK293^Cx43+^) with the parental cell line expressing very low levels of Cx43 (electronic supplementary material, figure S1*a*). Among the known targets of YBX1, we analysed the mRNA levels of matrix metalloproteinase (MMP)-13 [30], due to its known association with cardiac pathophysiology. Interestingly, significantly decreased levels of *MMP-13* were observed in Cx43-expressing cells (HEK293^Cx43+^; figure 2*g*), suggesting that the presence of Cx43 interferes with the expression of *MMP-13*. As expected, knockdown of YBX1 (electronic supplementary material, figure S3*g*) decreased the expression of *MMP-13,* but only when cells express low levels of Cx43 (HEK293^Cx43−^; figure 2*g*). According to our model, in the absence of Cx43, YBX1 is available to control the expression of *MMP-13*, which can be modulated by the knockdown of YBX1. By contrast, in HEK293^Cx43+^ cells where *MMP-13* expression is lower, knockdown of YBX1 was not able to further decrease mRNA levels of *MMP-13* (figure 2*g*). Altogether, we speculate that interaction of Cx43 with YBX1 affects its binding to DNA, thus hindering *MMP-13* transcription.

### 2.3. Importin-β participates in the transport of Cx43 to the nucleus

**Figure 3.**
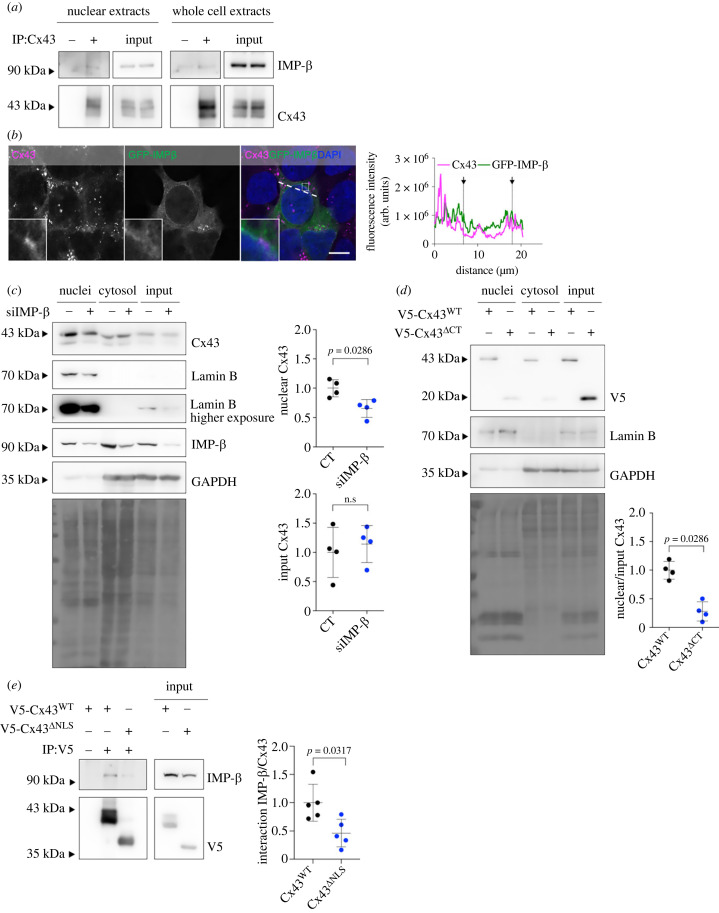
Translocation of Cx43 to the nucleus depends on Importin-β. (*a*) Cx43 was IP from purified nuclear or whole cell extracts, after which interaction between Cx43 and Importin-β was analysed by WB. (*b*) HEK293^Cx43+^ cells were transfected with GFP-Importin-β (GFP-IMP*β*) for 24 h, after which co-localization between Cx43 and GFP-Importin-β was analysed by confocal microscopy. Line profile plots depict the intensity distribution of green and magenta channels along the dashed white lines in the merged image. Arrows in co-localization plots indicate the margins of the nucleus. Inset images display a magnified view of the boxed region. Scale bars, 10 µm. (*c*) siRNA-mediated knockdown of Importin-β was performed in HEK293^Cx43+^ cells, for 48 h. Sucrose cushion-based nuclei purification was performed, followed by WB analysis of Cx43. Graphs depict quantification of nuclear and input Cx43 levels, normalized by total protein levels (Ponceau staining; *n* = 4 biological replicates). (*d*) Levels of nuclei-localized Cx43^WT^ or Cx43^ΔCT^ were assessed by WB in purified nuclei. Graphs depict quantification of nuclear Cx43, normalized for transfected Cx43 (input levels; *n* = 4 biological replicates). (*e*) V5 was IP from whole-cell extracts of HEK293^Cx43−^ cells transfected with V5-tagged full-length (Cx43^WT^) or NLS-deleted Cx43 (Cx43^ΔNLS^) for 24 h. Graph shows interaction levels of importin-β with Cx43 (V5) assessed by WB (*n* = 5 biological replicates). n.s: non-significant.

One of the proteins identified in our proteomic analysis, Ran GTPase-activating protein 1 (RanGAP1), is known to contribute to maintaining the GDP/GTP gradient across the nuclear envelope, regulating Ran-dependent import and export of nuclear proteins [31,32]. Given the role of Importin-β as a partner for Ran-mediated transport of nuclear cargo, we hypothesized that a similar mechanism could drive the trafficking of Cx43 into the nucleus. To address that, we started by validation of the MS data, demonstrating that Cx43 interacts and co-localizes with GFP-RanGAP1 and Importin-β in whole-cell and purified nuclear extracts (figure 3*a*,*b*; electronic supplementary material, figure S3*h–i*). Importantly, both chemical inhibition of Importin-β mediated nuclear import with ivermectin (Ive) [33] (electronic supplementary material, figure S4*a–c*) and Importin-β knockdown (figure 3*c*) significantly decreased the levels of nuclei-localized Cx43, suggesting that Importin-β plays a role in the transport of Cx43 to the nucleus. Accordingly, inhibition of Importin-β-mediated trafficking by raising intracellular calcium levels with ionomycin or thapsigargin, decreased nuclear Cx43 levels (electronic supplementary material, figure S4*d–e*).

Next, we sought to investigate which domains on Cx43 mediate the binding to Importin-β. Since the C-terminal tail of Cx43 contains multiple domains involved in protein–protein interactions, we started by assessing the impact of deleting the entire C-terminal region of Cx43 (K258stop, Cx43^ΔCT^) upon the interaction with Importin-β. Expression of Cx43^ΔCT^ prevented the interaction between Cx43 and Importin-β and the nuclear localization of Cx43 (figure 3*d*; electronic supplementary material, figure S4*f*), indicating that the C-terminal tail of Cx43 harbours the binding site for Importin-β. Given that Importin-β-dependent nuclear import relies on the recognition of NLS motifs on cargo proteins, we conducted a bioinformatic analysis to predict the existence of NLS signals on the Cx43 sequence [34]. The NLS mapper tool identified two sequential putative NLS sites with a cutoff of 3.1, from the amino acid 237 to 291 (electronic supplementary material, figure S4*g*), within the C-terminus of Cx43. Hence, we generated a Cx43 construct harbouring a deletion from amino acid 237 to 291, to mutate the putative NLS motif of Cx43 (Cx43^ΔNLS^). Our results (figure 3*e*; electronic supplementary material, figure S4*h*) show that the deletion of the putative NLS motif decreases the interaction of Cx43 with Importin-β, which was accompanied by a reduced localization of Cx43^ΔNLS^ to the nucleus. Altogether, these data suggest that an NLS-mediated binding of Cx43 to Importin-β at least partially mediates its transport into the nucleus.

### 2.4. PKA and Wnt signalling activation drive Cx43 translocation to the nucleus

**Figure 4.**
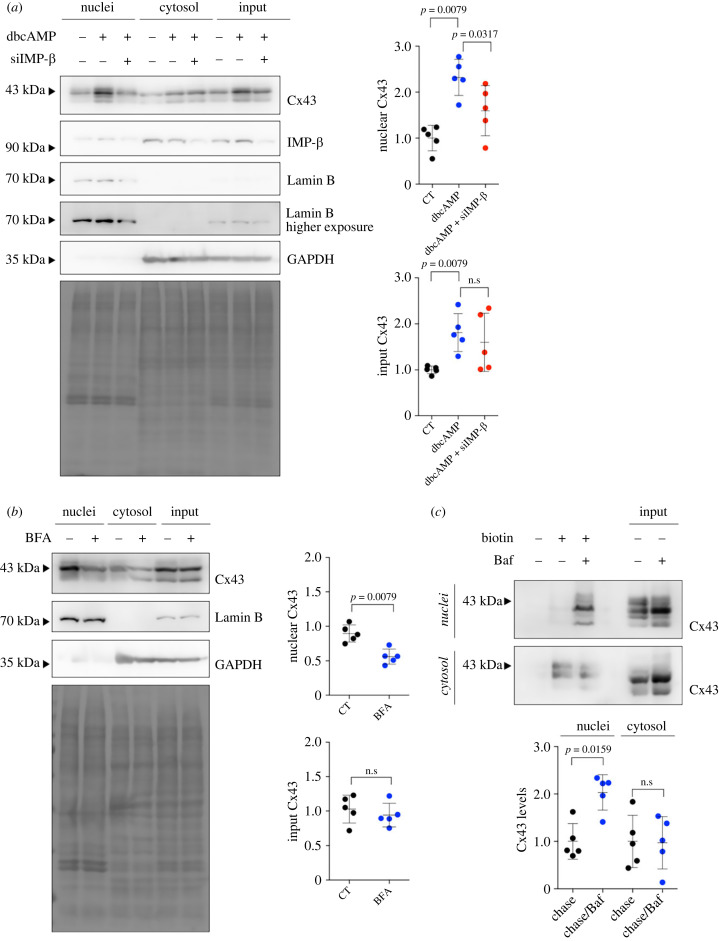
Activation of PKA promotes Importin-β-mediated transport of Cx43 to the nucleus. (*a*) siRNA-mediated knockdown of Importin-β was performed in HEK293^Cx43+^ cells, for 48 h. In the last 24 h, cells were treated with 1 mM of dbcAMP. Levels of Cx43 in purified nuclear extracts were evaluated by WB. Graphs depict quantification of nuclear and input Cx43 levels, normalized by total protein levels (Ponceau staining; *n* = 5 biological replicates). (*b*) Nuclear Cx43 levels were assessed after treatment with 5 µM Brefeldin A (BFA) for 2 h and sucrose cushion-based nuclei purification. Graphs depict quantification of nuclear and input Cx43 levels, normalized by total protein levels (Ponceau staining; *n* = 5 biological replicates). (*c*) Cell-surface biotinylation was performed for 30 min, at 4°C. After quenching of unbound biotin, cells returned to the 37°C incubator for 4 h, in the presence or absence of 100 nM Bafilomycin A1 (Baf). Nuclei and cytosolic fractions were purified following a sucrose cushion-based protocol. Cx43 levels were evaluated by WB after pull-down of biotinylated proteins on neutravidin beads (*n* = 5 biological replicates). n.s: nonsignificant.

At this stage, our data demonstrate that Cx43 can be transported to the nucleus by an Importin-β-mediated manner. However, the stimuli regulating the interaction between Cx43 and Importin-β and its consequences upon trafficking of full-length Cx43 to the nucleus remain unknown. Given that the activation of PKA-mediated signalling was previously shown to enhance the translocation of Cx43 to the nucleus [20], we next sought to assess whether this pathway impacts Importin-β-dependent transport of Cx43. Our results confirmed that PKA activation with dibutyryl cAMP (dbcAMP) increased the levels of nuclear Cx43 (figure 4*a*), which was prevented by Importin-β knockdown, suggesting that the transport of Cx43 to the nucleus elicited by PKA activation depends on Importin-β. In agreement, dbcAMP promoted the interaction between Cx43 and Importin-β (electronic supplementary material, figure S5*a*).

More recent studies suggested that the trafficking of Cx43 to the nucleus can be mediated by β-catenin, which is also a well-established interacting partner of Cx43, namely in the heart [21,35]. Thus, we proceeded to unravel whether β-catenin constitutes an additional regulatory player in Importin-β-mediated transport of Cx43. We started by demonstrating that activation of Wnt signalling using the agonist WAY-262611 increased the localization of Cx43 to the nucleus (electronic supplementary material, figure S5*b*), which was accompanied by an increased interaction between Cx43 and Importin-β (electronic supplementary material, figure S5*a*). On the other hand, when we activated the Wnt pathway using the glycogen synthase kinase 3-β (GSK3-β) inhibitor BI5521, we still observed an increase in the levels of nuclei-localized Cx43 (electronic supplementary material, figure S5*c*), without changes in the binding of Cx43 to Importin-β (electronic supplementary material, figure S5*a*). This observation is in line with previous evidence showing that GSK3-β inhibition promotes nuclear retention of β-catenin, rather than enhancing its nuclear import [36]. Consistently, BI5521 increased the levels of nuclear Cx43 even after the knockdown of Importin-β (electronic supplementary material, figure S5*d*).

Altogether, our results suggest that PKA and Wnt pathways modulate Cx43 trafficking to the nucleus, through a mechanism involving Importin-β.

### 2.5. Nuclear Cx43 is partially derived from the plasma membrane

Although our findings have shed some light into the mechanism underlying the transport of Cx43 into the nucleus, the origin of nuclear Cx43 remains unclear, that is, whether it derives from the ER, the plasma membrane or any subcellular structure *en route* to the plasma membrane. The ‘diffusion–retention’ model for INM targeting conceives that newly synthesized proteins freely diffuse within the continuous ER/ONM, before accumulating at the INM, which is limited by the size of nucleoplasmic domains of cargo proteins that should be below 60 kDa [37,38]. To evaluate whether full-length Cx43 can reach the INM through this pathway, we fused a GFP and a mCherry molecule to the N-terminal of Cx43, thus increasing the steric hindrance of the putative nucleoplasmic domain of Cx43. Our results (electronic supplementary material, figure S6*a*) show that nuclear localization of GFP-mCherry-Cx43 was not affected, which suggests that free diffusion is not responsible for nuclear Cx43 targeting.

After synthesis, Cx43 is co-translationally inserted into ER membranes and transported to the Golgi, where Cx43 oligomerizes into hexameric channels before trafficking to the plasma membrane [39]. Hence, we proceeded to address whether blockade of the ER exit or the secretory pathway affected the trafficking of Cx43 to the nucleus. First, we evaluated the levels of nuclear Cx43 after fusion with an ER-retention signal (Cx43^RRRRISLS^), or knockdown of the chaperone ERp29, required for ER exit of Cx43 [40,41]. Our results show that both strategies resulted in a decreased localization of Cx43 to the nucleus (electronic supplementary material, figure S6*b–c*). Next, we inhibited the secretory pathway with Brefeldin A (BFA) [42], which also caused a significant reduction in the levels of nuclear Cx43 (figure 4*b*). Importantly, BFA treatment decreased the interaction of Cx43 with Importin-β (electronic supplementary material, figure S6*d*), suggesting that Cx43 binding to Importin-β occurs following the Cx43 release from the ER. To specifically follow the trafficking of Cx43 after leaving the ER, we co-expressed an ER-resident bacterial biotin ligase (BirA-ER) together with a chimeric Cx43 fused with an AviTag peptide (Cx43^AviTag^) that can be specifically recognized and biotinylated by BirA-ER. Our data show that Cx43 biotinylated at the ER can be found in purified nuclei 16 h following biotin chase, which could be prevented by BFA (electronic supplementary material, figure S6*e*), thus reinforcing the idea that nuclear Cx43 derives from a post-ER population.

Since BFA treatment is known to impair the trafficking of Cx43 to the cell surface, reducing the amount of Cx43 at the plasma membrane [43], the decreased levels of nuclear Cx43 following BFA treatment may result from a depletion of the plasma membrane pool of Cx43. In fact, multiple plasma membrane channels and receptors have been reported to reach the nucleus following ligand-induced internalization [44]. Thus, we next sought to investigate whether nuclear Cx43 can also originate from the plasma membrane. To address that, we performed cell surface biotinylation at 4°C (pulse), after which intracellular routes of biotin-labelled proteins were chased for 4 h at 37°C. Our results demonstrate that biotinylated Cx43 is found in the nucleus after 4 h, particularly when protein degradation was inhibited with Bafilomycin A1 (Baf; figure 4*c*). Since Baf does not prevent internalization of Cx43, these data suggest that at least part of the nuclear Cx43 originates from the plasma membrane.

Overall, our results are consistent with a model in which Cx43 exits the ER and reaches the nucleus by an Importin-β-dependent mechanism, either directly after trafficking through the Golgi, or following targeting to the plasma membrane.

### 2.6. Cx43 modulates the gene expression landscape of cultured cells

**Figure 5.**
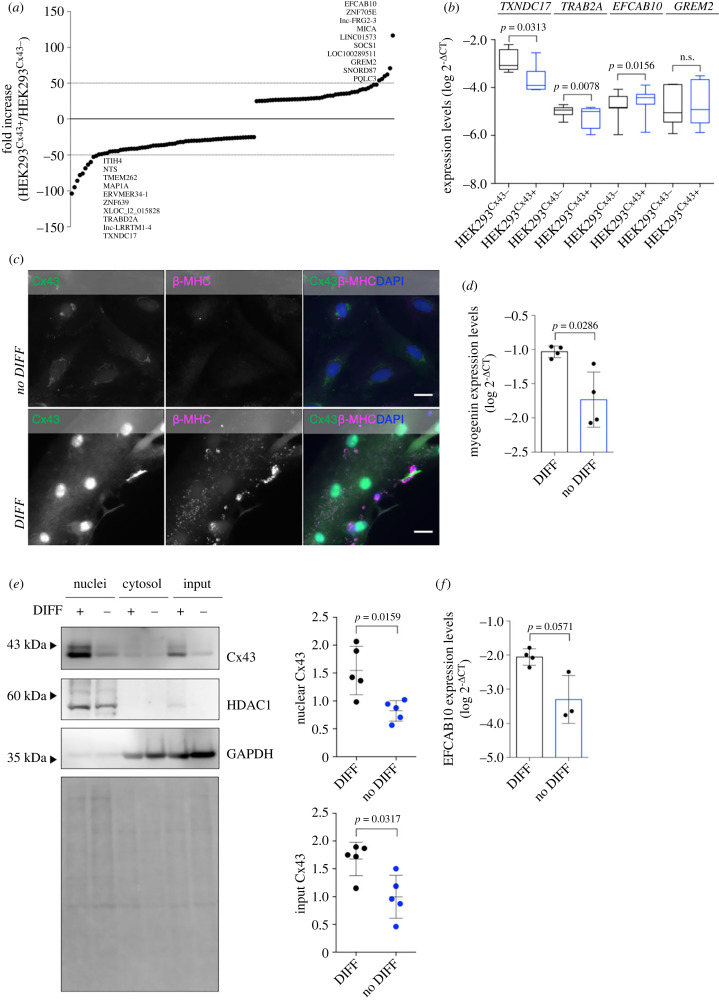
Cx43 modulates the cellular transcriptome. (*a*) A genome-wide profiling of mRNA was performed following microarray hybridization in HEK293^Cx43+^ and HEK293^Cx43−^ cells. RNA expression levels were quantified as log2 and analysed as fold change levels of HEK293^Cx43+^ over HEK293^Cx43−^ cells (*n* = 4 biological replicates). Graph depicts the fold change values for the 119 differentially expressed genes. Genes with higher fold change levels (top 10 upregulated and 10 downregulated) are detailed on graph. (*b*) mRNA expression levels of *TXNDC17*, *TRABD2A*, *EFCAB10* and *GREM2* were assessed by RT-qPCR in HEK293^Cx43+^ and HEK293^Cx43−^ cells. Results were normalized using *r18s* levels. Values are expressed as log2^−ΔCT^ (*n* = 6–8 biological replicates). n.s: non-significant. (*c*) Differentiation of H9c2 cells into myotubes was induced for 7 days (DIFF). Cells were fixed and stained for Cx43 (green; antibody AB0016, SICGEN) and β-MHC (magenta) before widefield fluorescence microscopy imaging. Nuclei were stained with DAPI. Scale bars, 20 µm. (*d*) Expression of *myogenin* was analysed by RT-qPCR in differentiated (DIFF) and undifferentiated H9c2 cells (no DIFF). Results were normalized using *r18s* and *GAPDH* levels. Values are expressed as log2^−ΔCT^ (*n* = 4 biological replicates). (*e*) Differentiation of H9c2 cells into myotubes was induced for 7 days. Sucrose cushion-based nuclei purification was performed, followed by WB analysis of Cx43. Graphs depict quantification of nuclear and input Cx43 levels, normalized by total protein levels (Ponceau staining; *n* = 5 biological replicates). (*f*) Expression of *EFCAB10* was analysed by RT-qPCR in differentiated (DIFF) and undifferentiated H9c2 cells (no DIFF). Results were normalized using *r18s* and *GAPDH* levels. Values are expressed as log2^−ΔCT^ (*n* = 4 biological replicates).

Mounting evidence has associated the expression of Cx43 with profound effects on the cellular transcriptome [13,45]. Here, we hypothesized that the presence of Cx43 in the nucleus determines these alterations. Hence, we proceeded to evaluate the impact of Cx43 levels on the cellular transcriptome. For that, we performed genome-wide profiling of mRNA in HEK293^Cx43+^ and HEK293^Cx43−^ cells, which revealed 119 differentially expressed genes, suggesting that the presence of Cx43 has an impact on gene transcription (figure 5*a*; electronic supplementary material, table S2). Despite our pipeline being designed for the detection of mRNA, the differential expression of some long non-coding RNA (lncRNAs) was also observed, suggesting that Cx43 may also modulate the expression of non-coding RNA sequences. Nonetheless, additional validation studies are required to confirm this association.

Sixty-eight genes were downregulated and 51 were upregulated in HEK293^Cx43+^ cells. Enrichment analysis linked differentially expressed genes to the transforming growth factor (TGF)-β signalling and immunity-related pathways (electronic supplementary material, figure S7*a–d*), implicating Cx43-regulated gene expression in disease pathophysiology. Based on the selection criteria of fold change greater than 50 and the biological relevance of the genes, we validated some of the hits by quantitative real-time PCR (RT-qPCR): *TXNDC17*, *TRABD2A*, *EFCAB10* and *GREM2*. The results depicted in figure 5*b* show that *TXNDC17* and *TRABD2A* are significantly downregulated, while *EFCAB10* is significantly upregulated in HEK293^Cx43+^ cells, validating the reliability of our profiling approach.

Cx43 was previously demonstrated to be required for the initial phases of normal myogenesis, before Cx43-dependent GJ communication being downregulated in mature fibres [46,47]. Hence, to provide additional clues about the biological relevance of our findings, we induced myogenic differentiation of H9c2 cardiomyoblasts, a well-established model to obtain terminally differentiated post-mitotic cells [48]. For that, we subjected the cells to low-serum containing medium, which gave rise to multinucleated cells, harbouring positive staining for the sarcomeric myosin heavy chain protein (β-MHC), present in adult muscle cells, and higher expression levels of myogenin, a marker of terminally differentiated myoblasts (figure 5*c*,*d*). Strikingly, we observed an accumulation of Cx43 in the nucleus following differentiation (figure 5*c*,*e*). Trafficking of Cx43 to myotubes nuclei was promoted by PKA activation, while BFA treatment significantly decreased the levels of nuclear Cx43 (electronic supplementary material, figure S7*e–f*), corroborating our initial findings in HEK293 cells and suggesting that the mechanisms underlying the transport of Cx43 to the nucleus are conserved in physiologically relevant cell types expressing endogenous Cx43.

Lastly, we hypothesized that the accumulation of Cx43 in the nucleus during H9c2 myotube differentiation could impact gene expression in this model and contribute to regulate myotube function. Given that calcium signalling is a critical regulator of myocyte homeostasis, we analysed the mRNA levels of *EFCAB10* that encodes for a calcium-binding protein, and which levels are significantly elevated in Cx43-expressing cells (figure 5*b*). Data in figure 5*f* show that *EFCAB10* is upregulated in differentiated H9c2 cells, suggesting that nuclear Cx43 can modulate gene transcription during myogenic differentiation.

Altogether, our data demonstrate that Cx43 significantly impacts gene expression, which can be mediated by the presence of Cx43 in the cell nucleus. Additional studies are required to further establish this association, which may have implications for muscle cell function, namely by regulating calcium signalling.

### 2.7. Cx43 assembles into functional channels at the nuclear envelope of cardiac cells

Having established the localization and trafficking of Cx43 into the nucleus, we proceeded to investigate whether Cx43 channels at the nuclear envelope are functional. To assess whether Cx43 hemichannels mediate the transport of small molecules across the nuclear envelope, we performed fluorescence recovery after photobleaching (FRAP) using calcein-AM dye, an approach widely used to investigate Cx43 channel-mediated intercellular communication that can also be employed to study nuclear dynamics [49,50]. Our results show that fluorescence recovery to the nuclei is faster in HEK293^Cx43+^ cells, when compared with cells expressing low levels of Cx43 (HEK293^Cx43−^; electronic supplementary material, figure S8). Interestingly, pre-treatment with the GJ inhibitor 18-β-glycyrrhetinic acid (18GA) prevented this effect and the recovery rate of Cx43-positive nuclei was similar to that of HEK293^Cx43−^, suggesting that nuclear Cx43 hemichannels are active (electronic supplementary material, figure S8).

**Figure 6.**
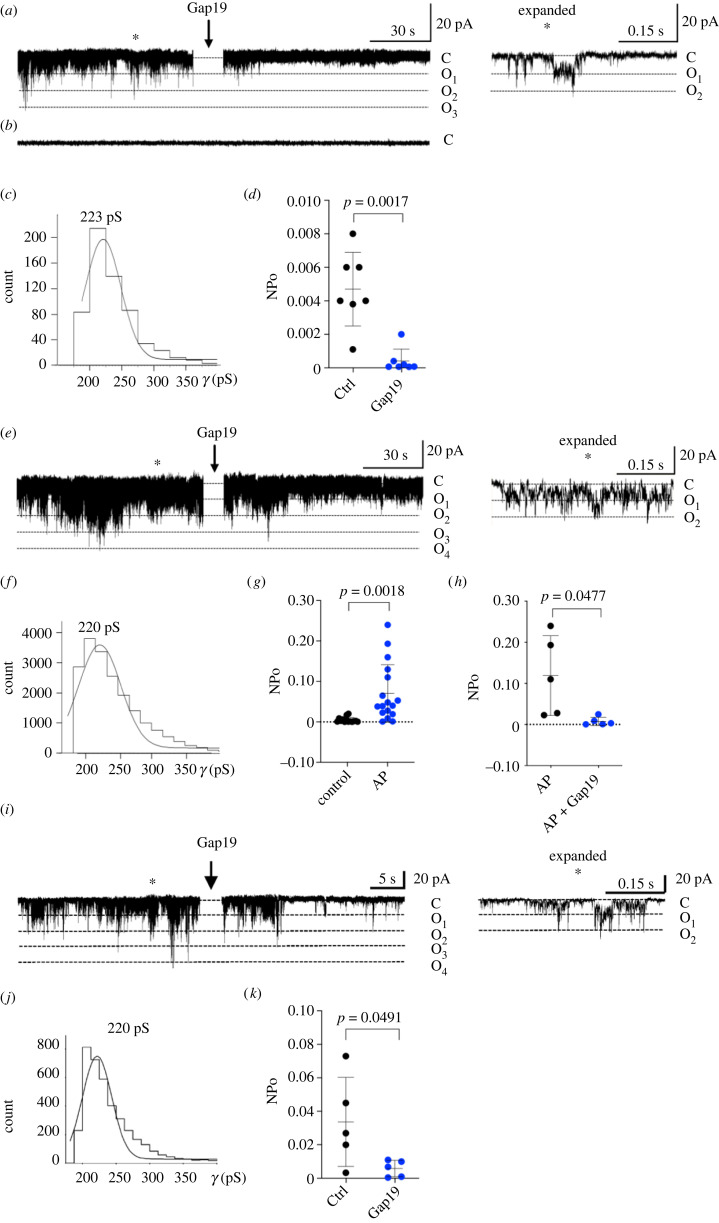
Outer nuclear membrane current recordings demonstrate Cx43 hemichannel opening activity. (*a*) Example trace recorded from HEK293^Cx43+^ nuclei demonstrating spike-like channel opening activity occurring from a closed state C to distinct open levels marked O_1_–O_3_. (*b*) Control recording in HEK293^Cx43−^ nuclei showed no activity. (*c*) Histogram analysis of unitary current activity indicated a conductance of approximately 223 pS that is typical for Cx43 hemichannels. Consequently, O_1_–O_3_ levels on the trace correspond to 223, 446 and 669 pS conductance respectively. The expanded trace right illustrates stacked opening events resulting from simultaneously occurring channel openings reaching O_2_ conductance levels. The spiking activity disappeared after bath application of the Cx43 hemichannel inhibitor Gap19 (100 µM). (*d*) Dot plot illustrating average effect of Gap19 on nominal open probability (NPo) of hemichannel opening. (*e*) Hemichannel activity was strongly increased in HEK293^Cx43+^ nuclei dephosphorylated by alkaline phosphatase, which was significantly decreased by Gap19. The expanded trace illustrates stacked openings after dephosphorylation. (*f*) Histogram analysis indicating activity of approximately 220 pS unitary conductance. (*g*) Summary scatter plot illustrating that dephosphorylation with alkaline phosphatase (AP, 100 units; 20 U µl; pH 7.4) significantly increased hemichannel activity (*n* = 15 biological replicates). (*h*) Summary data illustrating strong inhibition of increased activity by Gap19 (*n* = 5 biological replicates). (*i*) Example trace recorded from adult mouse ventricular cardiomyocyte nuclei demonstrating spike-like channel openings similar to those recorded from HEK293^Cx43+^ nuclei. Gap19 (100 µM) inhibited the channel activity. The expanded trace on the right shows stacked openings reaching O_2_ conductance levels. (*j*) Histogram analysis of unitary current activity in cardiomyocyte nuclei indicated a conductance of approximately 220 pS. (*k*) Summary plot showing inhibitory effect of Gap19 on NPo of Cx43 hemichannel activity registered in cardiomyocyte nuclei (*n* = 5 biological replicates).

To document hemichannel opening in detail, we performed patch-clamp on freshly isolated nuclei from HEK293^Cx43+^ and HEK293^Cx43−^ cells in ONM-attached recording mode. Fast spiking channel activity was recorded in approximately 20% of ONM patches of HEK293^Cx43+^ nuclei when positive voltages ≥30 mV were applied to the pipette with respect to the bath. Active patches showed a large unitary conductance in the range of approximately 220 pS and multiple stacked openings could be observed, indicating the simultaneous opening of up to 3 hemichannels per patch (figure 6*a*). Histogram analysis reported a unitary conductance of approximately 223 ± 7 pS (*n* = 7 biological replicates) that is characteristic for Cx43 hemichannels (figure 6*c*) [51–53]. The fast-spiking current activity was strongly diminished after application of the specific Cx43 hemichannel inhibitor Gap19 (figure 6*a*,*d*), while no currents were observed in HEK293^Cx43−^ cells (figure 6*b*). Dephosphorylation is known to increase Cx43 hemichannel activity, as observed in mitochondrial Cx43 hemichannels [27]. In line with this, a highly significant (15-fold) increase in hemichannel activity in the ONM of HEK293^Cx43+^ was observed upon alkaline phosphatase treatment (figure 6*e–g*). Unitary conductance remained at approximately 220 pS after alkaline phosphatase treatment, and Gap19 again strongly inhibited (14-fold) the current activity (figure 6*f–h*).

We further looked into the nucleus of adult cardiomyocytes, whose nuclear envelope structure and function have been demonstrated to impact the development and progression of multiple cardiac diseases [54]. Patch-clamp experiments revealed the presence of active Cx43 hemichannels in freshly isolated nuclei from adult mouse ventricular cardiomyocytes. As demonstrated in figure 6*i–k*, fast spiking channel activity recorded in ONM-attached configuration from cardiomyocyte nuclei was strikingly similar to that observed in recordings from HEK293^Cx43+^ nuclei (compare to figure 6*a–d*). Active patches from cardiomyocyte nuclei showed multiple stacked open levels indicating the simultaneous opening of up to 4 hemichannels in the patch (figure 6*i*). Histogram analysis of unitary current activity recorded from cardiomyocyte nuclei (figure 6*j*) demonstrated a conductance in the range of approximately 220 pS as previously described for plasma membrane Cx43 hemichannels in cardiomyocytes [51,52]. The fast-spiking current activity recorded from the ONM of cardiomyocyte nuclei was inhibited after application of Gap19 (figure 6*i*,*k*), suggesting involvement of Cx43 hemichannels.

Overall, our data indicate that Cx43 assembles into functional channels at the nuclear envelope of cultured cells and primary adult cardiomyocytes. Further studies are required to elucidate the possible role of these channels in regulating ion homeostasis and gene transcription in the nucleus.

## 3. Discussion

**Figure 7.**
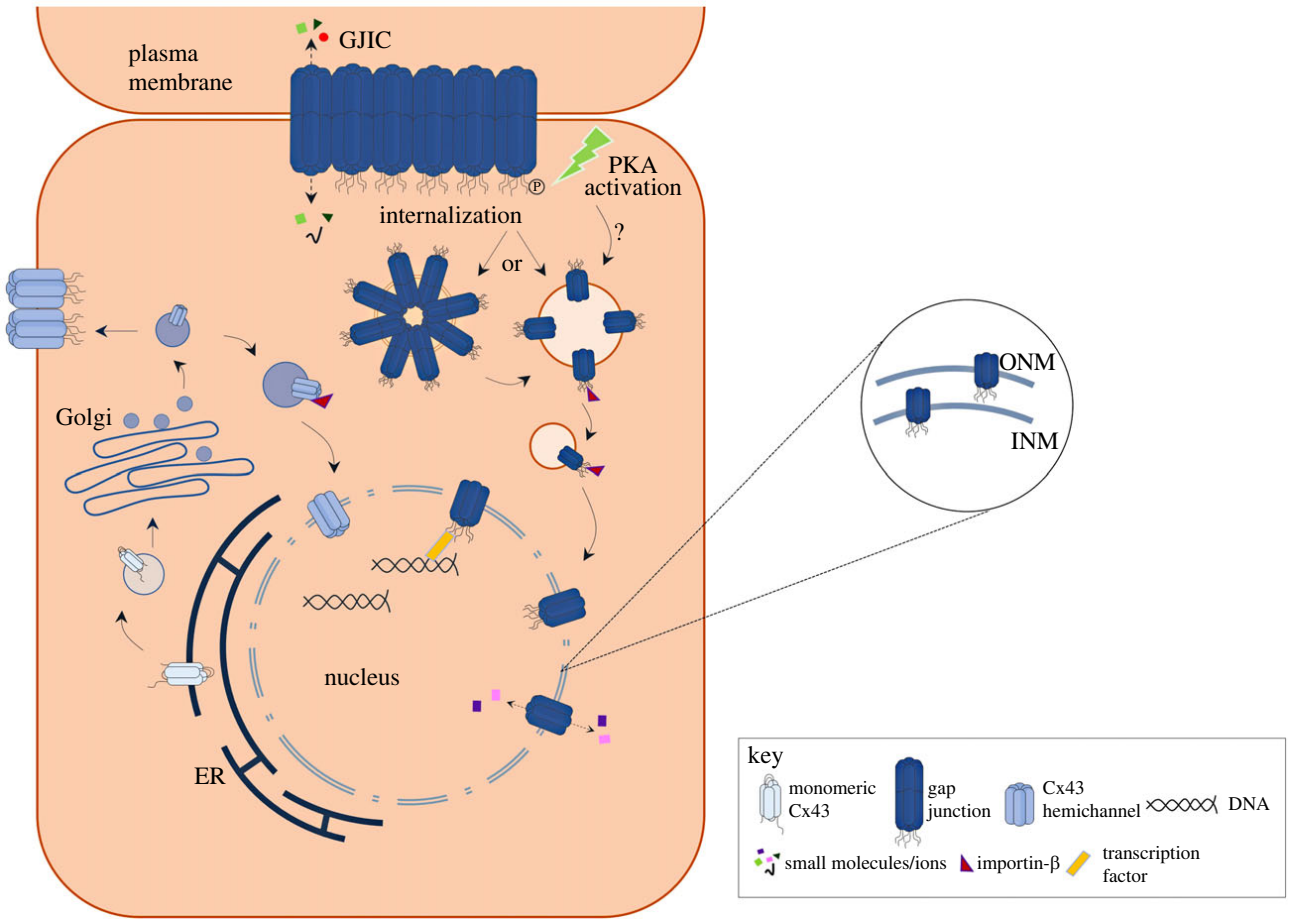
Working model for the transport and function of nuclear Cx43. Cx43 exits the ER and reaches the nucleus after interaction with Importin-β, either directly after trafficking through the Golgi, or following targeting to the plasma membrane, which can be modulated by activation of PKA signalling. Once at the nucleus, Cx43 hemichannels may participate in nucleocytoplasmic shuttling of small molecules and ions. Nuclear Cx43 can interact with transcription factors, likely contributing to regulation of gene expression. Inset depicts localization of Cx43 channels at the nuclear envelope—Cx43 can localize at both the INM and ONM.

In recent years, Cx43 has been associated with multiple GJ channel-independent functions, many of which are closely related to the presence of Cx43 in intracellular compartments, including the mitochondria and the nucleus [55]. Here, using a comprehensive approach and numerous complementary techniques, we consistently show that endogenous full-length Cx43 localizes at the nuclear envelope in multiple cell lines of different origins, adult primary cardiomyocytes, as well as in rat and human cardiac tissue, likely forming active hexameric channels. Importantly, we demonstrate that the presence of Cx43 affects gene expression, namely in HEK293^Cx43+^ and cardiac cells. Although we cannot discard the possibility that other populations of Cx43, namely GJ or hemichannels at the cell surface, can also contribute to the effects observed upon gene expression, considering all the data gathered in this study, we speculate that nuclear Cx43 plays an important biological role in shaping transcriptomic profile, either by acting as a nuclear ion channel or by interacting with proteins with transcription factor activity (working model, figure 7).

A few previous studies have shown that Cx43 can be found in the nucleus; however, most of these reports mainly aimed to unravel the biological implications of such nuclear localization, overlooking a meticulous biochemical characterization that allows, for example, to exclude contamination with lipid rafts or ER, in which Cx43 also localizes. Hence, we used thorough experimental approaches in HEK293^Cx43+^ and in various cell lines with endogenous Cx43 expression, including subcellular fractionation that yielded pure nuclear fractions with little contamination by other intracellular membranes, and high-resolution optical and electron microscopy to show that Cx43 localizes at the nuclear membrane, both INM and ONM.

Although we acknowledge that parental HEK293 cells express low levels of Cx43, the HEK293^Cx43+^ cell line, with stable physiological overexpression of Cx43, represents a suitable tool to study the signalling pathways, localization, trafficking and function of nuclear Cx43 [7]. To demonstrate that the results obtained with HEK293^Cx43+^ are not cell type-specific or an artefact of overexpression, key observations were further validated in different cell models with endogenous Cx43 expression. Despite the intrinsic limitations of using a cell line, we believe that studies comparing HEK293^Cx43+^ cells and the parental HEK293^Cx43−^ cell line, with lower levels of Cx43, constitute appropriate models to mimic what happens during pathological conditions, in which remodelling and downregulation of Cx43 have been very often demonstrated.

To unveil some clues concerning the role of nuclear Cx43, we performed an unbiased MS analysis of nuclear-specific Cx43 interactors. This approach also allowed us to shed some light on the mechanisms underlying the transport of nuclear Cx43, namely by identifying RanGAP1, involved in Importin-α/β-dependent trafficking [32]. In a previous proteomic study from our laboratory, we have also identified the interaction between Cx43 and Importin-β in rat hearts, suggesting that this mechanism is conserved in cardiac cells, in which the transport of Cx43 into the nucleus may have relevant (patho)physiological consequences [9]. Importin-β has been implicated in the trafficking of multiple members of the EGFR family of receptors, harbouring functional NLS motifs [22,23,56]. Similarly, in the present work, we observed that Cx43 interacts with Importin-β in both total and nuclear extracts and that Cx43 transport to the nucleus was impaired following the knockdown of Importin-β. In addition to the genetic manipulation of Importin-β levels, we demonstrate that chemical inhibition of Importin-β-mediated transport with Ive also decreases the levels of nuclear Cx43. Although initially described as a specific Importin-β inhibitor, Ive was recently demonstrated to induce DNA damage and cellular toxicity in multiple myeloma cells, which may indirectly contribute to affect the trafficking of Cx43 to the nucleus [33,57]. Importantly, we observed an increased translocation of Lamin B to the nucleus following Ive treatment, which is a hallmark of nuclear envelope changes upon DNA damage. Future research aiming to explore the impact of cytotoxicity on the transport of Cx43 to the nucleus will be important to further understand the pathophysiological implications of nuclear Cx43.

Although truncation of the entire Cx43 C-terminus and the predicted NLS motif of Cx43 prevented interaction with Importin-β and nuclear localization of Cx43, it is important to acknowledge that both sequences are considerably large and harbour binding sites for other Cx43 interactors, as well as residues that can be subjected to post-translational modifications, which can also modulate the subcellular trafficking of Cx43, thus indirectly contributing to impact on the nuclear Cx43 levels. Moreover, we cannot exclude that interaction of Importin-β with Cx43 requires an additional intermediate interacting protein, or the presence of other sorting motifs distinct from NLS within the Cx43 C-tail, including arginine (Arg) clusters. Importantly, an Arg-based sequence on SUN2 was demonstrated to mediate coat protein complex (COP)-I-mediated retrograde trafficking from the Golgi to the ER, thereby maintaining a pool of SUN2 that can be further delivered to the nuclear envelope [58]. Given that the C-terminus of Cx43 harbours a putative COP-I retention motif (R^374^PR^376^), this pathway may also contribute to nuclear Cx43 targeting [59]. In agreement, when we blocked Cx43 trafficking through the Golgi using BFA or depleting ERp29, the amount of Cx43 found in the nucleus was decreased.

Furthermore, our results show that Importin-β-mediated transport of Cx43 to the nucleus is enhanced upon PKA activation, in both dividing HEK293 cells and terminally differentiated H9c2 myotubes. Although Cx43 can be subjected to phosphorylation by PKA in multiple residues (S364, S365, S373, S369), it is currently unclear whether these post-translational modifications modulate the interaction between Cx43 and Importin-β [60]. Since Cx43 can be phosphorylated following activation of other kinases, including PKC and calcium/calmodulin-dependent protein kinases (CaMKs), we cannot discard the possibility that activation of other signalling pathways may also modulate Cx43 transport to the nucleus, e.g. under pathological conditions.

Our data demonstrate that nuclear Cx43 is partially derived from the plasma membrane pool of Cx43. Since both PKA and Wnt signalling activation have been associated with the enhanced formation of GJ channels [61,62], we speculate that PKA/Wnt-mediated accumulation of Cx43 at the cell surface precedes its trafficking to the nucleus. Further investigation is required to elucidate how internalized Cx43, which classically follows the endo-lysosomal pathway, can be diverted and recognized by the nuclear import machinery. Moreover, it remains unclear whether the nuclear transport of Cx43 requires the interaction with both Importin-β and β-catenin, or if these two interacting partners play independent trafficking roles. Given that interaction between Cx43 and β-catenin is known to occur at the intercalated discs of cardiomyocytes, activation of Wnt signalling may likely control the disruption of these junctional complexes with the consequent translocation of both proteins into the nucleus [35]. It is also important to acknowledge the different effects observed following treatment with a GSK3-β inhibitor, which despite increasing the levels of nuclear Cx43, did not affect its interaction with Importin-β. Blocking of GSK3-β was previously related with impaired export of nuclear β-catenin, rather than affecting its import [36]. Therefore, we can anticipate that interaction between Cx43 and β-catenin is maintained within the nucleus and may control the export of Cx43.

Interestingly, our results demonstrate that the levels of nuclear Cx43 are significantly increased during differentiation of H9c2 cardiomyoblasts into myotubes. Since activation of Wnt/β-catenin signalling has been previously reported to regulate myotube formation, it is possible that an increased translocation of Cx43 to the nucleus during myogenic differentiation is driven by this pathway [63], which awaits further experimental confirmation.

After establishing the presence of hexameric structures at the nuclear membrane, we next aimed to establish whether nuclear Cx43 forms functional channels that can mediate the flux of ions and small molecules. Patch-clamp and FRAP analyses indicate that nuclear Cx43 assembles into functional hemichannels, with unitary conductance levels similar to those found at the plasma membrane [51–53]. Additionally, we show that nuclear hemichannel opening activity is inhibited by the Cx43 hemichannel blocker Gap19 and enhanced by alkaline phosphatase, implicating phosphorylation of Cx43 as a gatekeeper of nuclear Cx43 hemichannels, as is the case for plasmalemmal hemichannels and GJ channels. GJ-mediated passage of small metabolites and second messengers has been previously reported to alter the subcellular localization and/or the activity of transcription factors, thereby impacting the cellular transcriptome [64]. Since we provide preliminary evidence that Cx43 assembles into hexameric channels at the nuclear envelope, namely of cardiomyocytes, the passage of ions and small molecules through nuclear Cx43 hemichannels may indirectly affect gene transcription. Further experiments are required to establish the biological role of nuclear Cx43 hemichannels, and the mechanistic basis of how nuclear Cx43 may modulate cardiac cell function in health and disease conditions.

Consistent with previous studies reporting altered transcriptome and proteome profiles in Cx43-expressing cells, our results show that 119 genes are differentially expressed in the presence of Cx43 [13,45]. However, it remains unclear whether Cx43 modulates gene transcription through a direct or an indirect process, and whether the nuclear Cx43 pool is the main contributor to this effect. Previous studies have shown that the truncated isoform GJA1–20k can directly control the transcription of N-cadherin during neural crest cell migration, through binding to basic transcription factor 3 and polymerase II [16]. Here, we identify the interaction of Cx43 with proteins involved in the regulation of gene transcription and mRNA processing, such as Ca^2+^-regulated heat stable protein 1 (CARHSP1), SNW1, myelin expression factor 2 (MYEF2) and YBX1 that can also shape the cellular transcriptome. In agreement, our results suggest that the presence of Cx43 modulates the impact of YBX1 on the expression of *MMP-13*, which might be extended to other YBX1 targets. Additional chromatin immunoprecipitation (ChIP) studies are required to establish the role of nuclear Cx43 in the modulation of transcription factor activity. Although our data point to a role of nuclear Cx43 in mediating gene transcription, further in-depth investigation is required, including Cx43 knock-out studies and the functional consequences of altering the transport of full-length Cx43 to the nucleus.

Although we cannot establish a causal relationship between the accumulation of nuclear Cx43 and the increased expression of myogenin, our findings agree with previous reports, which also demonstrated that depletion of Cx43 decreases both the expression of myogenin and myoblast fusion [46]. Importantly, in terminally differentiated myotubes, the accumulation of Cx43 in the nucleus also correlates with an increased expression of *EFCAB10*, one of the genes that is upregulated in Cx43-expressing cells. Given the function of EFCAB10 as a calcium-binding protein, and the fact that proteins involved in calcium handling are more expressed in differentiated H9c2 cells [65], the presence of Cx43 in the nucleus of differentiated myotubes may contribute to improve calcium handling. It can be further speculated that nuclear Cx43 levels modulate the transcriptome landscape of myotubes, with important implications upon regulation of muscle contractility. Nonetheless, additional studies addressing the impact of nuclear Cx43 on the expression of other calcium-handling regulators, beyond *EFCAB10*, are needed.

It is well established that subcellular distribution of Cx43, namely its accumulation at the plasma membrane forming GJ, is different according to the cell origin and pathological condition. Not surprisingly, the levels of nuclear Cx43 vary among the different biological samples we used in our study, including multiple cell lines of different origins (HEK293, MCEC, NRK, H9c2 and C33a), primary adult cardiomyocytes, and human and rat heart cell types. Furthermore, it is expectable that the amount of Cx43 at the nucleus changes in (patho)physiological conditions, implicating nuclear Cx43 in the onset and development of diseases.

In the present study, we focused on Cx43 that is the most broadly expressed family member in humans. However, other connexins may also localize to the nucleus, forming functional channels. Since connexin channel composition determines selective permeability to small solutes and second messengers, channels formed by different connexins may play distinct regulatory roles in nuclear ion transport and/or gene expression. Given that dysfunction of other connexin family members is also associated with human pathologies, the non-canonical functions of connexins in the nucleus may constitute key components in disease pathophysiology, not only in the heart, but also in the nervous system, the skin, or the cochlea [66]. Hence, our findings provide an important toolkit to develop novel mechanistic-based therapeutic strategies targeting connexin-related disorders, based on Importin-β-mediated transport and/or the domains on Cx43 responsible for its trafficking to the nucleus.

Overall, the results obtained in this work enabled a comprehensive characterization of the biological role of Cx43 localized at the nuclear envelope of cardiac cells, as well as the signals and mechanisms underlying the transport of nuclear Cx43. Grounded on data showing that Cx43 accumulates in the nucleus following myogenic differentiation, we speculate that changes in nuclear Cx43 levels during cardiac development and disease contribute to modulate the gene expression profile and pathological phenotype in the heart, which requires additional experimental validation. Therefore, by shedding light into the biological roles of nuclear Cx43, our study can open new avenues to design novel therapeutic strategies aiming at targeting Cx43-mediated gene expression.

## 4. Material and methods

### 4.1. Antibodies and chemicals

Antibodies against Calnexin (AB0041), Cx43 (AB0016; epitope within C-terminus of Cx43, aa. 237–382), GAPDH (AB0049) and GFP (AB0020) were purchased from SICGEN (Cantanhede, Portugal). Antibodies against Caveolin-1 (sc-894), Emerin (H12, sc-25284), Flotillin-1 (sc-25506), p21 (sc-756), HA (F-7, sc-7392), hnRNPA2B1 (sc-374053) and Importin-β/karyopherin-β1 (H-7; sc-137016) were obtained from SCBT (Heidelberg, Germany), and against Lamin B (NA12) were from ORP (San Diego, CA, USA). Antibodies against p53 (ab131442) and Nedd4 (ab14592) were from abcam (Cambridge, UK), against SART1 (H9092-BO1P) were from Abnova (Taipei City, Taiwan), and against ERp29 (HPA039456) were from Atlas Antibodies (Bromma, Sweden). Antibodies against SNW1 (AB_2619117), YBX1 (AB_2619228), HDAC1 (AB_2722192) and Lamin A/C (AB_2618203) were from DSHB (Iowa University, IA, USA). Antibodies against Cx43 (610062; epitope within C-terminus of Cx43, aa. 252–270) were from BD Biosciences (Franklin Lakes, NJ, USA), and against MYH7/β-MHC (A7564) were from ABclonal (Wuhan, China). Antibodies against myc (13-2500), Cx43 (710700; epitope within C-terminus of Cx43, aa. 237–382) and non-phosphorylated Cx43 (CX-1B1, 13–8300) were from Thermo Fisher Scientific (Waltham, MA, USA). Unless stated otherwise, all chemicals were obtained from Sigma-Aldrich (St Louis, MO, USA).

### 4.2. Cell culture

Human embryonic kidney (HEK293) cells, C33a, H9c2 and normal rat kidney epithelial cells (NRK) were obtained from ATCC and cultured in Dulbecco's modified Eagle medium (DMEM, Gibco, Thermo Fisher Scientific), supplemented with 10% FBS (Gibco, Thermo Fisher Scientific) and 1% penicillin/streptomycin (100 U ml^−1^ : 100 µg ml^−1^). Mouse cardiac endothelial cells (MCEC) were obtained from Dr Justin C. Mason (Imperial College London, London, UK) and maintained in DMEM with 10 mM HEPES, 5% FBS and 1% penicillin/streptomycin (100 U ml^−1^ : 100 µg ml^−1^). HL-1 mouse cardiomyocytes were obtained from Sigma-Aldrich and maintained in Claycomb medium, supplemented with 2 mM L-glutamine, 0.1 mM norepinephrine, 10% FBS and 1% penicillin/streptomycin (100 U ml^−1^ : 100 µg ml^−1^). All cell lines were maintained at 37°C under 5% CO_2_. For myotube differentiation studies, H9c2 cells at 70–80% confluency were cultured in DMEM supplemented with 1% FBS, 1% insulin-transferrin-selenium (ITS, Sigma-Aldrich) and 1% penicillin/streptomycin for 7 days [48].

HEK293^Cx43+^ cell line was established as previously described [67]. Briefly, HEK293 parental cells (HEK293^Cx43−^) were transduced with the lentiviral vector pLenti6-prom-CMV-V5-C1-Cx43 (Cx43 of rat origin). After 1 day, 8 µg ml^−1^ blasticidin was added to select transduced cells. HEK293^Cx43+^ cell line stably expressing Cx43-APEX2 or Cx43^AviTag^ were established following the same protocol. Cell lines were not recently authenticated but are routinely tested for mycoplasma.

### 4.3. Primary cardiac cell cultures

Neonatal and adult rat ventricular myocytes were isolated from Wistar rats (P5 and 2- to 3-month-old males, respectively), obtained from our local breeding colony, as previously described [7,68]. Animals were handled according to European Union guidelines (2010/63/EU), approved by ORBEA-IBILI (permit 13/2015). For neonatal cell isolation, hearts were excised and subjected to 0.1% trypsin-EDTA digestion overnight, at 4°C, followed by a type II collagenase (Gibco, 75 U ml^−1^) digestion for 30 min, at 37°C. Tissue was mechanically dissociated in DMEM + 10% FBS and transferred through a screen (70 μm). Cells were recovered by centrifugation and pre-plated into 1% (w/v) gelatin-coated dishes for 3 h, before final plating of non-adherent cardiomyocytes in fibronectin-coated dishes. Cells were maintained in DMEM + 10% FBS and 1% penicillin/streptomycin (100 U ml^−1^ : 100 µg ml^−1^). Adult cardiomyocytes were isolated following perfusion of the heart with digestion buffer containing type II collagenase (Gibco, 54.2 U ml^−1^) and mechanical dissociation using forceps and plastic Pasteur pipettes. Ca^2+^ tolerant viable cardiomyocytes were selected and plated in laminin-coated dishes (BD Biosciences) with DMEM + 5% FBS, 1% ITS and penicillin/streptomycin (100 U ml^−1^ : 100 µg ml^−1^). All cultures were maintained at 37°C under 5% CO_2_.

### 4.4. Human samples

Biopsy specimens were obtained from human hearts of patients that required aortic valve replacement due to severe aortic stenosis, and had concomitantly a bulged interventricular septum, causing some degree of left ventricular outflow tract obstruction, which was the indication for performing septal myectomy (Center of Cardiothoracic Surgery, Centro Hospitalar e Universitário de Coimbra (CHUC), Coimbra, Portugal). All patients gave their informed consent for surgery and had granted permission for use of their medical records for research purposes. This form has been approved by the Ethics Committee of our institution.

### 4.5. Cell treatments

5 µM Brefeldin A (BFA, Sigma-Aldrich) was used to inhibit the secretory pathway and 100 nM Bafilomycin A1 (Baf, SCBT) was used to inhibit lysosomal degradation. To activate PKA signalling, 1 mM dbcAMP (SCBT) was used for 24 h. Wnt signalling was activated by 2 µM WAY-262611 (WAY, SCBT) for 6 h, or by inhibiting GSK-3*β* with 30 µM BI5521 (opnMe, Boehringer Ingelheim) for 1 h. To modulate Importin-β-dependent transport, 50 µM Ivermectin (ive, SCBT) for 6 h, 0.5 µM ionomycin (iono, Sigma-Aldrich) or 250 nM thapsigargin (thaps, SCBT) for 1 h were used.

### 4.6. Plasmid constructions and cell transfection

The ER-retention motif RRRRISLS was subcloned into a pENTR-V5-Cx43 vector to generate Cx43^RRRRISLS^. Plasmids expressing ^GFP^Cx43, ^GFPmCherry^Cx43, Cx43^Avi^ and BirA-NLS were generated by cloning the appropriate cDNA into a pENTR vector containing the indicated tags. Plasmids expressing Cx43^ΔNLS^ were generated by amplifying cDNA encoding the first 236 and last 91 amino acids of rat Cx43, followed by a second PCR to unite the fragments; and finally cloned into a pENTR V5 vector. NLS-EGFP was kindly provided by Rob Parton (Addgene #67652) [69], mEmerald-Nesprin3-C-18 from Michael Davidson (Addgene #54203), Emerin pEGFP-N2 (588) from Eric Schirmer (Addgene #61985) [70], pDESTmycYBX1 from Thomas Tuschl (Addgene #19878) [71], pEGFP-C2 RanGAP from Mary Dasso (Addgene #13378) [72], pEGFP-N1 full length Importin- β was a gift from Patrizia Lavia (Addgene #106941) [73], pcDNA3 Connexin43-GFP-APEX2 (Addgene #49385) and pDisplay-BirA-ER (Addgene #20856) from Alice Ting [74,75]. Experiments were performed 24 h after transient transfection of cells with Lipofectamine 2000 (Thermo Fisher Scientific), according to the manufacturer.

### 4.7. siRNA-mediated protein knockdown

Transfections were performed with Lipofectamine 2000 (Thermo Fisher Scientific) according to the manufacturer. Briefly, experiments were performed 48 h after siRNA (20 nM final concentration) transfection. Non-targeting sequences (Ambion, Thermo Fisher Scientific) were used as controls. siRNA against human YBX1 (siGENOME (4904) siRNA SMARTpool) were obtained from Horizon Discovery (Dharmacon, Lafayette, CO, USA). siRNA against ERp29 (sc-60599) and IMP*β* (sc-35736) were obtained from SCBT.

### 4.8. Isolation of nuclei from cultured cells and primary cardiomyocytes

Cells were resuspended in homogenization buffer (0.25 M sucrose, 25 mM KCl, 5 mM MgCl_2_, 1 mM dithiothreitol (DTT), 50 mM Tris-HCl pH 7.4, supplemented with protease inhibitors—protease inhibitor cocktail (Roche, Basel, Switzerland) and 2 mM sodium orthovanadate) and passed 5–6 times through a 23-gauge needle. Samples were centrifuged at 500*g*, for 10 min to obtain crude nuclear pellets; supernatants were collected as the cytosolic soluble fraction. Crude nuclear pellets were resuspended in nuclei homogenization buffer supplemented with 1% Triton X-100 (TX100), after which they were added on top of a 1 ml 1.8 M sucrose cushion (1.8 M sucrose, 5 mM MgAc_2_, 0.1 mM EDTA, 10 mM Tris-HCl pH 7.4) and centrifuged for 1 h, at 27 500*g*. Purified nuclear pellets were resuspended in nuclei homogenization buffer supplemented with 1% TX100 and 0.2% SDS.

For solubility assays, crude nuclear pellets were resuspended in nuclear homogenization buffer supplemented with either 7 M urea, 50 mM or 250 mM NaCl and 1% Triton X-100, followed by vigorous vortexing and centrifuging at 27 500*g* for 15 min at 4°C, to obtain soluble (supernatant) and insoluble nuclear fractions (pellet).

Total protein content was quantified using the DC™ Protein Assay (Bio-Rad, Hercules, CA, USA), before western blot (WB) analysis.

### 4.9. Isolation of nuclear envelopes

Purification of nuclear envelopes was performed as previously described [76]. All steps were performed at 4°C. All solutions were supplemented with protease inhibitors (protease inhibitor cocktail (Roche) and 2 mM sodium orthovanadate) and 2 mM DTT. Briefly, cells were resuspended in homogenization buffer (0.3 M sucrose, 60 mM KCl, 2 mM EDTA, 0.5 mM EGTA, 10 mM HEPES pH 7.4), and passed 5–6 times through a 23-gauge needle. Samples were centrifuged at 1000*g*, for 10 min. Pellets were resuspended in SHKM buffer (0.25 M sucrose, 25 mM KCl, 5 mM MgCl_2_, 50 mM HEPES pH 7.4), passed through a 23-gauge needle and centrifuged for 20 min, at 4000*g*. Pellets were resuspended in 1.85 M sucrose, overlaid with 2.15 M and 2.8 M sucrose layers, and centrifuged for 60 min, at 82 000*g*. Nuclei were collected from the 2.8 and 2.15 M layers, diluted 10-fold in sucrose-free homogenization buffer and pelleted at 4000*g*, for 20 min. Nuclear pellets were further subjected to two rounds of digestion with DNase and RNase in 10% SHM buffer (0.3 M sucrose, 2 mM MgCl_2_, 0.5 mM CaCl_2_, 10 mM HEPES pH 7.4) for 20 min, and centrifuged at 4000*g*. Supernatants were collected (nucleoplasm fraction); pellets containing nuclear envelopes were resuspended in 10% SHM supplemented with 300 mM NaCl, overlaid with 30% SHM buffer (0.9 M sucrose) and centrifuged for 30 min, at 6000*g*. Purified nuclear envelopes were recovered in the pellets.

### 4.10. Trypsin resistance assay

Trypsin resistance assay was performed in nuclei isolated from HEK293^Cx43+^ cells and purified on a 1.8 M sucrose cushion. Nuclei were incubated with 0.01 µg µl^−1^ trypsin for 10 min at 4°C. Enzymatic reaction was stopped by the addition of Laemmli buffer and denatured at 95°C, for 5 min before WB analysis.

### 4.11. Alkaline phosphatase dephosphorylation assay

Alkaline phosphatase dephosphorylation assay was performed in purified nuclei from HEK293^Cx43+^ cells. Nuclear pellets (15 µg total protein) were lysed in RIPA buffer and further incubated with 30 U alkaline phosphatase for 1 h at 37°C. Enzymatic reaction was stopped by the addition of Laemmli buffer and denatured at 95°C, for 5 min before WB analysis.

### 4.12. Western blot analysis

Samples were separated by sodium dodecylsulfate polyacrylamide gel electrophoresis (SDS-PAGE) and transferred to nitrocellulose membranes. Membranes were blocked with 5% non-fat milk in Tris-buffered saline-Tween 20 (TBS-T; 20 mM Tris, 150 mM NaCl, 0.2% Tween 20, pH 7.6), probed with appropriate primary antibodies and horseradish peroxidase (HRP)-conjugated secondary antibodies. Proteins of interest were visualized by chemiluminescence using a LAS500 system (GE Healthcare, Chicago, IL, USA). Densitometric quantification was performed in unsaturated images using ImageJ (National Institutes of Health, Bethesda, MD, USA).

### 4.13. Immunofluorescence staining

Cells grown on fibronectin-coated coverslips were fixed in 4% paraformaldehyde (PFA) for 10 min and permeabilized with 0.5% TX100 for 10 min. In TX100 live extraction experiments, cells grown on fibronectin-coated coverslips were incubated with TX100 extraction buffer (3% TX100, 10 mM HEPES, 80 mM KCl, 16 mM NaCl, 1.5 mM MgCl_2_, 1 mM DTT, 30% glycerol, pH 7.9), for 5 min at 4°C, before fixation with 4% PFA for 10 min [77]. Rat and human heart tissues for immunostaining were harvested and embedded in optimal cutting temperature (OCT, Tissue-Tek, Sakura, Alphen aan den Rijn, The Netherlands). 10 µm heart slices were fixed in cold acetone for 20 min at −20°C. In all cases, specimens were blocked with 2.5% bovine serum albumin (BSA) for 20 min, followed by incubation with appropriate primary antibodies overnight, at 4°C. Alexa Fluor-conjugated secondary antibodies were incubated for 1 h, at room temperature. All solutions were made in 0.25% BSA. Nuclei were stained with DAPI (4′,6-diamidino-2-phenylindole). Specimens were mounted with Mowiol 4–88. For controls, primary antibodies were omitted. Images were analysed by confocal microscopy (Zeiss LSM 710, Carl Zeiss AG, Jena, Germany), or widefield fluorescence microscopy (Axio Observer.Z1, Carl Zeiss AG), as indicated, using a Plan-Apochromat 63x/1.4 Oil DIC M27 objective (Carl Zeiss AG).

### 4.14. Single-molecule localization microscopy

24 h after transfection with mEmerald-Nesprin3, MCEC cells were fixed, permeabilized and stained with primary antibodies against GFP (598, from MBL International (Woburn, MA, USA)) and against Cx43 (AB0016, from SICGEN), followed by secondary Alexa Fluor donkey anti-rabbit 647 (Thermo Fisher Scientific) and CF^TM^ 680 donkey anti-goat (Sigma-Aldrich). Samples were mounted in imaging buffer (200 U ml^−1^ glucose oxidase, 1000 U ml^−1^ catalase, 10% (w/v) glucose, 200 mM Tris-HCl pH 8.0, 10 mM NaCl and 50 mM cysteamine). Single-molecule localization microscopy (SMLM) was performed on a customized Leica SR GSD system using an HCX PL APO 160x/1.43 Oil CORR GSD objective, a 642 nm laser for excitation and a 405 nm laser for reactivation of fluorophores. For two-colour imaging, the fluorescence was split into two spectral channels with an Optosplit II image splitter and imaged side-by-side on an Andor iXon Ultra 897 camera. Ratiometric demixing of fluorophores was done in SplitViSu with subsequent drift correction and image reconstruction in SharpViSu [78,79].

### 4.15. Transmission electron microscopy and immunogold imaging

For immunogold labeling, cells were fixed for 30 min at room temperature with 2% PFA/0.2% glutaraldehyde in PHEM buffer (60 mM PIPES, 25 mM HEPES pH 7.0, 10 mM EGTA, 2 mM MgCl_2_). After rinsing in 50 mM glycine in PHEM buffer, samples were embedded in 10% gelatin in PBS, cut in small blocks and cryo-protected at 4°C (2.3 M sucrose) for 16 h. Blocks were mounted onto cryo-microtomy pins and frozen in liquid nitrogen. Ultra-thin cryo-sections (80 nm) were prepared in a Leica FC7/UC7, at −110°C with a diamond knife (Diatome, Switzerland) and picked up with a drop of 1% (w/v) methylcellulose/1.15 M sucrose in PHEM buffer. Sections were transferred to Formvar carbon-coated nickel grids, followed by immunolabelling in an automated system (EM IGL, Leica microsystems) at room temperature. Grids were incubated for 5 min in PBS (0.1 M, pH 7.4), quenched with 50 mM glycine in PBS for 10 min, followed by blocking for 30 min (#905.002, Aurion, Wageningen, The Netherlands). Samples were washed (2 × 5 min, PBS/BSA 0,1%), and incubated with rabbit anti-Cx43 antibodies (710700, Thermo Fisher Scientific, 1 : 30 in PBS/BSA 0,1%) for 2 h. After washing (4 × 5 min, PBS/BSA 0,1%), grids were incubated for 1 h with 10 nm gold conjugate goat anti-rabbit secondary antibodies (#806.011, Aurion, 1 : 5 in PBS/BSA 0,1%). After washing (4 × 5 min, PBS/BSA 0,1% and 4 × 5 min, PBS), specimens were stabilized for 5 min with 2% glutaraldehyde in PBS, and washed (2 × 5 min, PBS and 6 × 5 min, deionized water). Before observation, grids were incubated for 5 min on 2% uranyl acetate (pH 7.0) and transferred to a mixture of 1.8% methyl cellulose and 0.4% uranyl acetate (pH 4) on ice for 5 min. Excess of the viscous solution was drained and the grids were left to dry before imaging with a Tecnai G2 Spirit BioTwin (FEI, Hillsboro, OR, USA) TEM, operating at 120 kV, using an ORIUS SC1000 CCD camera (Gatan). Specimens incubated with secondary antibodies in the absence of anti-Cx43 antibodies were used as controls.

For Cx43-APEX2 staining, cells were fixed in 2% glutaraldehyde (in 100 mM sodium cacodylate with 2 mM CaCl_2_, pH 7.4) for 60 min. All steps until resin infiltration were performed at 4°C. Cells were rinsed in chilled buffer, treated in buffer containing 20 mM glycine for 5 min, followed by washing in chilled buffer. Cells were incubated for 5 min with a freshly diluted solution of 0.5 mg ml^−1^ 3,3′-diaminobenzidine (DAB) free base (Sigma-Aldrich), combined with 10 mM H_2_O^2^ in chilled buffer. Specimens were rinsed with chilled buffer and stained with 2% (w/v) osmium tetroxide (Electron Microscopy Sciences) for 30 min in chilled buffer. After rinsing, cells were incubated with 2% (w/v) uranyl acetate in ddH_2_O (Electron Microscopy Sciences) overnight. Cells were gently scraped off the dishes, resus­pended and centrifuged at 700*g* for 1 min to generate a cell pellet that was dehydrated in a graded ethanol series (50%, 75%, 90%, 95%, 100%, 100%, 100%), impregnated and embedded using an epoxy embedding kit (Fluka Analytical, Sigma-Aldrich). An ultramicrotome (EM UC6 + EM FC6) (Leica, Solms, Germany) was used to cut ultrathin sections, which were mounted on copper grids and stained with lead citrate (0.2%) for 7 min. Observation of the samples was carried out on a FEI-Tecnai G2 Spirit Bio Twin, at 100 kV.

### 4.16. Fluorescence recovery after photobleaching

HEK293^Cx43−^ and HEK293^Cx43+^ cells grown on fibronectin coated glass bottom dishes (Ibidi, Gräfelfing, Germany) were loaded with 0.5 µM calcein-acetoxymethyl ester (AM) for 30 min and incubated with DAPI for 10 min at 37°C, before FRAP. Where indicated, cells were pre-treated with 10 µM 18-β-glycyrrhetinic acid (18GA) for 30 min, at 37°C. Based on DAPI staining, individual nuclei were selected for photobleaching, using the 488 nm line of the argon laser of a Zeiss LSM 710 confocal microscope (Carl Zeiss AG). Fluorescence recovery analysis of bleached areas was performed for 330 s, with a measure every 15 s. Data were analysed using ImageJ.

### 4.17. Biotinylation of cell-surface proteins

Biotinylation assays were performed in HEK293^Cx43+^ cells using 0.5 mg ml^−1^ sulfo-NHS-SS-biotin [sulfosuccinimidyl-2-(biotinamido)ethyl-1,3-dithiopropionate] (Pierce, Thermo Fisher Scientific) in PBS containing 1 mM Ca^2+^ and 0.5 mM Mg^2+^ (PBS/Ca^2+^/Mg^2+^) for 30 min at 4°C. Excess biotin was quenched by PBS/Ca^2+^/Mg^2+^ supplemented with 100 mM glycine and washed with PBS/Ca^2+^/Mg^2+^. Cells were incubated in complete cell culture medium before being returned to a 37°C incubator for 2 h, to follow the intracellular trafficking of biotin-labelled proteins. After incubation, cells were harvested in nuclei homogenization buffer. Nuclei were purified, after which nuclear or cytosolic lysates were used for pull-down on neutravidin beads (Pierce, Thermo Fisher Scientific) for 2 h, at 4°C under agitation. Beads were washed with RIPA buffer (150 mM NaCl, 50 mM Tris-HCl, 1% NP-40 and 0.1% SDS, pH 7.5), and final pellets resuspended in Laemmli buffer and denatured at 95°C, for 5 min before SDS-PAGE and WB analysis.

### 4.18. Immunoprecipitation

Samples were prepared in RIPA buffer, containing protease inhibitors (protease inhibitor cocktail (Roche) and 2 mM sodium orthovanadate) and supplemented with 0.5% TX100. To IP Cx43, 0.25 µg goat anti-Cx43 (AB0016, SICGEN) were incubated with 500 µg total protein lysates overnight, at 4°C under agitation. 8 µg of dry protein G Sepharose beads (GE Healthcare) were added to the samples for 2 h, at 4°C under agitation. Beads were washed in RIPA buffer, followed by elution in Laemmli buffer and denaturation at 95°C, for 5 min.

For proteomic analysis, 3 mg total protein nuclear lysates were used for IP. Beads were washed three times in 1 ml trypsin digestion buffer (20 mM Tris-HCl pH 8.0, 2 mM CaCl_2_). MS analysis was performed in VIB Proteomics Core, VIB Center for Medical Biotechnology (Ghent, Belgium).

### 4.19. Database search for protein identification and quantification by mass spectrometry

Peptide samples were injected on liquid chromatography-MS/MS analysis and all raw files were searched together using the MaxQuant algorithm (version 1.6.3.4) with default search settings including a false discovery rate set at 1% on both the peptide and protein level. Spectra were searched against the human proteins in the Uniprot/Swiss-Prot database (database release version of January 2019 containing 20 398 human protein sequences; www.uniprot.org) supplemented with the recombinant Protein G sequence from *Staphylococcus* *aureus*. Enrichment analysis of identified nuclear Cx43 binding partners was performed with Enrichr [80].

### 4.20. RNA extraction and gene expression microarray technology

Total RNA was purified from HEK293^Cx43−^ and HEK293^Cx43+^ cells using the Qiagen RNeasy Mini Kit according to the manufacturer's protocol (Qiagen, Hilden, Germany). RNA integrity was determined using the RNA 6000 Nano LabChip Kit in an Agilent 2100 bioanalyzer (Agilent Technologies, Santa Clara, CA, USA). Only samples with RNA integrity number scores ≥8 were used and were then quantified using a NanoDrop 1000 spectrophotometer (Thermo Scientific, USA).

Labelling of RNA was performed using Low Input Quick Amp Labeling Kit, One Colour (Agilent Technologies), according to the manufacturer's instructions. 100 ng of total RNA was amplified and labelled with Cy3 dye. This amplification product was measured for quantity and dye incorporation using the Nanodrop 1000 and then hybridized to Agilent SurePrint G3 Human Genome Expression microarray V2, 8 × 60 K (Agilent Technologies). All microarray hybridizations were performed according to the manufacturer's instructions in the One-Colour Microarray-Based Gene Expression Analysis manual. The fluorescently labelled RNA was hybridized to the microarray at 65°C in a rotating oven (10 rpm). After 17 h, the arrays were washed consecutively in gene expression wash buffers. Fluorescence intensities were measured with an Agilent microarray scanner and extracted using Agilent Feature Extraction 10.7.3.1. For every gene, the mean and standard deviation were determined to calculate the interval using two standard deviations. The genes that exhibited non-overlapping two-standard intervals between each condition were selected, indicating potential differences between the groups. Following this selection process, a *p*-value was computed using a test for two independent samples on the set of chosen genes. Enrichment analysis of differentially expressed genes was performed with Enrichr [80].

### 4.21. Quantification of mRNA by quantitative real-time PCR

Validation of gene expression microarrays was performed by RT-qPCR. Total RNA was isolated from HEK293^Cx43−^ and HEK293^Cx43+^ cells with NZYol reagent (nzytech), according to the manufacturer. RNA concentrations were determined using a NanoDrop spectrophotometer (Thermo Fisher Scientific). Total RNA was reverse-transcribed using the NZY First-Strand cDNA Synthesis Kit (nzytech), and RT-qPCR reactions were performed using One-step NZY RT-qPCR Green kit, ROX plus (nzytech) in a StepOnePlus Real-Time PCR System (Applied Biosystems, Thermo Fisher Scientific). Gene expression changes were analysed using the StepOne software. The primers used are listed on table 1.

**Table 1.** List of primers used in the present study.

primer name	primer sequence
r18s FW	GTCTGCCCTATCAACTTTC
r18s RV	TTCCTTGGATGTGGTAGC
EFCAB10 (human) FW	GGAGGAAGTGAACAAGAGGATGA
EFCAB10 (human) RV	TTGCTGCTGACGTTACGAGA
EFCAB10 (rat) FW	TCAGGGACGAAGTGAACAAG
EFCAB10 (rat) RV	TTATCTTGCACTGCGATGCC
TRABD2A FW	ATGAGTCCCTGGAGCTGGTTC
TRABD2A RV	GGAATTCAGCTCGCTTTGTTGGG
TXNDC17 FW	ATGGCAAGACCATTTTCGCC
TXNDC17 RV	CTCGTACGACTGGTTCAGCC
GREM2 FW	CGCTTCTCTTATGGGCGTCT
GREM2 RV	CCAGAACATCCTGCAATGACG
MMP13 FW	CAGTTTGCAGAGCGCTACC
MMP13 RV	TTGCCAGTCACCTCTAAGCC
myogenin FW	GTCCCAACCCAGGAGATCATTT
myogenin RV	GACGTAAGGGAGTGCAGGTT
GAPDH FW	GACTTCAACAGCAACTCC
GAPDH RV	GCCATATTCATTGTCATACCA

### 4.22. Nuclear patch-clamp

Adult mouse ventricular cardiomyocytes were isolated as described previously [51,52]. Nuclei were freshly isolated from HEK293^Cx43−^, HEK293^Cx43+^ cells or cardiomyocytes as described by Mak *et al*. [81], using nuclei isolation buffer (150 mM KCl, 250 mM sucrose, 10 mM Tris pH 7.3, supplemented with protease inhibitor cocktail (Roche), 0.1 mM of Pefabloc SC (4-(2-aminoethyl)benzenesulfonyl fluoride hydrochloride, AEBSF, Roche) and 1.4 mM 2-mercaptoethanol). For nuclei isolation, HEK293 cells grown to 80% of confluency were harvested with 0.25% trypsin/EDTA (Gibco), washed twice with ice-cold PBS followed by centrifugation for 3 min at 1000 rpm at 4°C. Cell pellet of HEK293 cells or cardiomyocytes was resuspended in 1 ml of nuclei isolation solution, transferred to an ice-cold homogenizer (Kimble 885480-0020 Duall 1 ml), and subjected to 11 up-and-down strokes, yielding approximately 40% nuclei. Preparation was kept on ice and used within 2 h for patch-clamping the ONM.

Electrophysiological characterization of nuclear ion channel activity at single-channel level was done in the on-nucleus configuration, i.e. with the patch pipette in contact with the ONM that is normally exposed to the cytoplasm. Pipettes were pulled from borosilicate glass capillaries with filament (GB150F-10, Science products) and 7–10 MΩ resistance; stable seal resistances (1–10 GΩ) could be obtained lasting over tens of minutes. Currents were recorded with an EPC 7 PLUS amplifier (HEKA) in voltage-clamp mode, filtered by a 7-pole Bessel low-pass filter at 3 kHz cut-off frequency and digitized at 12 kHz using a NI USB-6221 data acquisition device (National Instruments) and WinWCP acquisition software developed by Dr J. Dempster (University of Strathclyde, UK). Both pipette and bath contained the same solution composed of 130 mM CsCl, 10 mM Na-aspartate, 0.26 mM CaCl_2_, 1 mM MgCl_2_, 2 mM ethylene glycol tetraacetic acid (EGTA), 5 mM tetraethylammonium (TEA)-Cl, 5 mM hydroxyethyl piperazineethanesulfonic acid (HEPES), 50 nM free [Ca^2+^] at pH 7.2. For dephosphorylation experiments, 15 µl of the nuclei suspension was incubated with 100 units of alkaline phosphatase (Roche) in 540 µl bath solution supplemented with 60 µl of 10X dephosphorylation buffer at pH 7.4 and 23°C for 30 min; control experiments were performed in solution without alkaline phosphatase and adjusted to pH 7.4. Single-channel analysis was done in Clampfit 10.7 (Molecular Devices). Unitary current activity was determined from all-point histograms of the current trace, which were fitted by a Gaussian distribution; unitary conductances were calculated from the elementary current transitions Δi as: *γ* = Δi/Vm. Channel activity is expressed as nominal open probability NPo, denoting the number of active channels in the patch multiplied by the open probability. The number of active channels in the patch was derived from stacked opening events resulting from coincident channel openings. For Cx43 hemichannel activity, the maximum number of active hemichannels in a patch was 3 (4 also occurred but were rare). NPo values were determined after setting the open–closed discriminator (threshold) half-way between the baseline and fully open state levels. NPo was calculated by dividing the time that the channel spent in the open state by the total time. Pooled data are expressed as mean ± SEM. Statistical comparisons were performed with two-tailed *t* tests for paired or unpaired data as appropriate.

### 4.23. Statistical analysis

Data are presented as individual data points with mean ± s.d. (*in vitro* experiments were performed with at least 3–5 independent biological replicates). Independent variables were analysed by Mann–Whitney test, whereas ANOVA (Tukey's *post hoc*) or Kruskal–Wallis (Dunn's *post hoc*) were used for multiple comparisons. In the absence of normal distribution (assessed by Shapiro–Wilk test), only non-parametric statistical tests were used. Analyses were performed with GraphPad Prism 6.01.

## Data Availability

Datasets generated during and/or analysed during the current study are available from the corresponding author on reasonable request. Raw data from gene expression microarrays are available at https://apps.uc.pt/mypage/faculty/fcaramelo/en/cardiacgenes. Supplementary material is available online [82].

## References

[1] Francastel C, Schübeler D, Martin DIK, Groudine M. 2000 Nuclear compartmentalization and gene activity. Nat. Rev. Mol. Cell Biol. **1**, 137-143. (10.1038/35040083) 11253366

[2] Schirmer EC, Gerace L. 2005 The nuclear membrane proteome: extending the envelope. Trends Biochem. Sci. **30**, 551-558. (10.1016/j.tibs.2005.08.003) 16125387

[3] Wilson KL, Berk JM. 2010 The nuclear envelope at a glance. J. Cell Sci. **123**, 1973-1978. (10.1242/jcs.019042) 20519579 PMC2880010

[4] Chang W, Worman HJ, Gundersen GG. 2015 Accessorizing and anchoring the LINC complex for multifunctionality. J. Cell Biol. **208**, 11-22. (10.1083/jcb.201409047) 25559183 PMC4284225

[5] Dauer WT, Worman HJ. 2009 The nuclear envelope as a signaling node in development and disease. Dev. Cell **17**, 626-638. (10.1016/j.devcel.2009.10.016) 19922868

[6] Prissette M et al. 2022 Disruption of nuclear envelope integrity as a possible initiating event in tauopathies. Cell Rep. **40**, 111249. (10.1016/J.CELREP.2022.111249) 36001963

[7] Martins-Marques T et al. 2020 EHD1 modulates Cx43 gap junction remodeling associated with cardiac diseases. Circ. Res. **126**, E97-E113. (10.1161/CIRCRESAHA.119.316502) 32138615

[8] Martins-Marques T. 2021 Connecting different heart diseases through intercellular communication. Biol. Open **10**, bio058777. (10.1242/bio.058777) 34494646 PMC8443862

[9] Martins-Marques T, Anjo SI, Pereira P, Manadas B, Girão H. 2015 Interacting network of the gap junction (GJ) protein connexin43 (Cx43) is modulated by ischemia and reperfusion in the heart. Mol. Cell. Proteomics **14**, 3040-3055. (10.1074/mcp.M115.052894) 26316108 PMC4638045

[10] Martins-Marques T, Ribeiro-Rodrigues T, Batista-Almeida D, Aasen T, Kwak BR, Girao H. 2019 Biological functions of connexin43 beyond intercellular communication. Trends Cell Biol. **29**, 835-847. (10.1016/j.tcb.2019.07.001) 31358412

[11] Gago-Fuentes R, Fernández-Puente P, Megias D, Carpintero-Fernández P, Mateos J, Acea B, Fonseca E, Blanco FJ, Mayan MD. 2015 Proteomic analysis of connexin 43 reveals novel interactors related to osteoarthritis. Mol. Cell. Proteomics **14**, 1831-1845. (10.1074/mcp.m115.050211) 25903580 PMC4587334

[12] Martins-Marques T et al. 2020 Myocardial infarction affects Cx43 content of extracellular vesicles secreted by cardiomyocytes. Life Sci. Alliance **3**, e202000821. (10.26508/LSA.202000821) 33097557 PMC7652393

[13] Iacobas DA, Urban-Maldonado M, Iacobas S, Scemes E, Spray DC. 2003 Array analysis of gene expression in connexin-43 null astrocytes. Physiol. Genomics **15**, 177-190. (10.1152/physiolgenomics.00062.2003) 12928503 PMC2651830

[14] Tarzemany R, Jiang G, Jiang JX, Larjava H, Häkkinen L. 2017 Connexin 43 hemichannels regulate the expression of wound healing-associated genes in human gingival fibroblasts. Sci. Rep. **7**, 14157. (10.1038/s41598-017-12672-1) 29074845 PMC5658368

[15] Kardami E, Dang X, Iacobas DA, Nickel BE, Jeyaraman M, Srisakuldee W, Makazan J, Tanguy S, Spray DC. 2007 The role of connexins in controlling cell growth and gene expression. Prog. Biophys. Mol. Biol. **94**, 245-264. (10.1016/j.pbiomolbio.2007.03.009) 17462721

[16] Kotini M, Barriga EH, Leslie J, Gentzel M, Rauschenberger V, Schambon A, Mayor R. 2018 Gap junction protein Connexin-43 is a direct transcriptional regulator of N-cadherin *in vivo*. Nat. Commun. **9**, 3846. (10.1038/s41467-018-06368-x) 30242148 PMC6155008

[17] Epifantseva I, Xiao S, Baum RE, Kléber AG, Hong TT, Shaw RM. 2020 An alternatively translated connexin 43 isoform, gja1-11k, localizes to the nucleus and can inhibit cell cycle progression. Biomolecules **10**, 473. (10.3390/biom10030473) 32244859 PMC7175147

[18] Varela-Eirin M, Varela-Vazquez A, Rodríguez-Candela Mateos M, Vila-Sanjurjo A, Fonseca E, Mascareñas JL, Eugenio Vázquez M, Mayan MD. 2017 Recruitment of RNA molecules by connexin RNA-binding motifs: implication in RNA and DNA transport through microvesicles and exosomes. Biochim. Biophys. Acta Mol. Cell Res. **1864**, 728-736. (10.1016/j.bbamcr.2017.02.001) 28167212

[19] Martins-Marques T, Costa MC, Catarino S, Simoes I, Aasen T, Enguita FJ, Girao H. 2022 Cx43-mediated sorting of miRNAs into extracellular vesicles. EMBO Rep. **23**, e54312. (10.15252/EMBR.202154312) 35593040 PMC9253745

[20] Chen X et al. 2016 Dynamic changes in protein interaction between AKAP95 and Cx43 during cell cycle progression of A549 cells. Sci. Rep. **6**, 21224. (10.1038/srep21224) 26880274 PMC4754773

[21] Hou X, Khan MRA, Turmaine M, Thrasivoulou C, Becker DL, Ahmed A. 2019 Wnt signaling regulates cytosolic translocation of connexin 43. Am. J. Physiol. Regul. Integr. Comp. Physiol. **317**, R248-R261. (10.1152/ajpregu.00268.2018) 31067079

[22] Wang YN, Hung MC. 2012 Nuclear functions and subcellular trafficking mechanisms of the epidermal growth factor receptor family. Cell Biosci. **2**, 13. (10.1186/2045-3701-2-13) 22520625 PMC3418567

[23] Giri DK, Bartholomeusz G, Hung M-C, Ali-Seyed M, Wang S-C, Ling P, Li L-Y, Lee D-F. 2005 Endosomal transport of ErbB-2: mechanism for nuclear entry of the cell surface receptor. Mol. Cell. Biol. **25**, 11 005-11 018. (10.1128/MCB.25.24.11005-11018.2005) PMC1316946 16314522

[24] Nili E et al. 2001 Nuclear membrane protein LAP2beta mediates transcriptional repression alone and together with its binding partner GCL (germ-cell-less). J. Cell Sci. **114**, 3297-3307. (10.1242/jcs.114.18.3297) 11591818

[25] Say YH, Hooper NM. 2007 Contamination of nuclear fractions with plasma membrane lipid rafts. Proteomics **7**, 1059-1064. (10.1002/pmic.200600849) 17351887

[26] Miro-Casas E et al. 2009 Connexin43 in cardiomyocyte mitochondria contributes to mitochondrial potassium uptake. Cardiovasc. Res. **83**, 747-756. (10.1093/cvr/cvp157) 19460776

[27] Gadicherla AK et al. 2017 Mitochondrial Cx43 hemichannels contribute to mitochondrial calcium entry and cell death in the heart. Basic Res. Cardiol. **112**, 27. (10.1007/s00395-017-0618-1) 28364353

[28] Zhong X et al. 2022 ERK/RSK-mediated phosphorylation of Y-box binding protein-1 aggravates diabetic cardiomyopathy by suppressing its interaction with deubiquitinase OTUB1. J. Biol. Chem. **298**, 101989. (10.1016/j.jbc.2022.101989) 35490780 PMC9163515

[29] Dumont AA, Dumont L, Berthiaume J, Auger-Messier M. 2019 p38*α* MAPK proximity assay reveals a regulatory mechanism of alternative splicing in cardiomyocytes. Biochim. Biophys. Acta Mol. Cell Res. **1866**, 118557. (10.1016/j.bbamcr.2019.118557) 31505169

[30] Ibrahim WN, Doolaanea AA, Bin Abdull Rasad MSB. 2018 Effect of shRNA mediated silencing of YB-1 protein on the expression of matrix collagenases in malignant melanoma cell *in vitro*. Cells **7**, 7. (10.3390/cells7010007) 29320405 PMC5789280

[31] Lui K, Huang Y. 2009 RanGTPase: a key regulator of nucleocytoplasmic trafficking. Mol. Cell. Pharmacol. **1**, 148-156. (10.4255/mcpharmacol.09.19) 20300488 PMC2839366

[32] Hutten S, Flotho A, Melchior F, Kehlenbach RH. 2008 The Nup358-RanGAP complex is required for efficient importin α/β-dependent nuclear import. Mol. Biol. Cell **19**, 2300. (10.1091/MBC.E07-12-1279) 18305100 PMC2366868

[33] Wagstaff KM, Sivakumaran H, Heaton SM, Harrich D, Jans DA. 2012 Ivermectin is a specific inhibitor of importin *α*/β-mediated nuclear import able to inhibit replication of HIV-1 and dengue virus. Biochem. J. **443**, 851. (10.1042/BJ20120150) 22417684 PMC3327999

[34] Kosugi S, Hasebe M, Tomita M, Yanagawa H. 2009 Systematic identification of cell cycle-dependent yeast nucleocytoplasmic shuttling proteins by prediction of composite motifs. Proc. Natl Acad. Sci. USA **106**, 10 171-10 176. (10.1073/PNAS.0900604106) 19520826 PMC2695404

[35] Shaw RM, Fay AJ, Puthenveedu MA, von Zastrow M, Jan YN, Jan LY. 2007 Microtubule plus-end-tracking proteins target gap junctions directly from the cell interior to adherens junctions. Cell **128**, 547-560. (10.1016/j.cell.2006.12.037) 17289573 PMC1955433

[36] Jamieson C, Sharma M, Henderson BR. 2011 Regulation of β-catenin nuclear dynamics by GSK-3*β* involves a LEF-1 positive feedback loop. Traffic (Copenhagen, Denmark) **12**, 983-999. (10.1111/J.1600-0854.2011.01207.X) 21496192

[37] Katta SS, Smoyer CJ, Jaspersen SL. 2014 Destination: inner nuclear membrane. Trends Cell Biol. **24**, 221-229. (10.1016/j.tcb.2013.10.006) 24268652

[38] Ungricht R, Klann M, Horvath P, Kutay U. 2015 Diffusion and retention are major determinants of protein targeting to the inner nuclear membrane. J. Cell Biol. **209**, 687-704. (10.1083/jcb.201409127) 26056139 PMC4460150

[39] Thévenin AF, Margraf RA, Fisher CG, Kells-Andrews RM, Falk MM. 2017 Phosphorylation regulates connexin43/ZO-1 binding and release, an important step in gap junction turnover. Mol. Biol. Cell **28**, 3595-3608. (10.1091/mbc.E16-07-0496) 29021339 PMC5706988

[40] Das S et al. 2009 ERp29 restricts connexin43 oligomerization in the endoplasmic reticulum. Mol. Biol. Cell **20**, 2593-2604. (10.1091/mbc.E08-07-0790) 19321666 PMC2682600

[41] Su V, Nakagawa R, Koval M, Lau AF. 2010 Ubiquitin-independent proteasomal degradation of endoplasmic reticulum-localized connexin43 mediated by CIP75. J. Biol. Chem. **285**, 40 979-40 990. (10.1074/jbc.M110.170753) PMC3003397 20940304

[42] Nebenfuhr A. 2002 Brefeldin A: deciphering an enigmatic inhibitor of secretion. Plant Physiol. **130**, 1102-1108. (10.1104/pp.011569) 12427977 PMC1540261

[43] Thomas T. 2005 Mechanisms of Cx43 and Cx26 transport to the plasma membrane and gap junction regeneration. J. Cell Sci. **118**, 4451-4462. (10.1242/jcs.02569) 16159960

[44] Carpenter G, Liao HJ. 2013 Receptor tyrosine kinases in the nucleus. Cold Spring Harb. Perspect. Biol. **5**, a008979. (10.1101/cshperspect.a008979) 24086039 PMC3783051

[45] Olk S, Turchinovich A, Grzendowski M, Stühler K, Meyer HE, Zoidl G, Dermietzel R. 2010 Proteomic analysis of astroglial connexin43 silencing uncovers a cytoskeletal platform involved in process formation and migration. GLIA **58**, 494-505. (10.1002/glia.20942) 19795503

[46] Araya R, Eckardt D, Maxeiner S, Krüger O, Theis M, Willecke K, Sáez JC. 2005 Expression of connexins during differentiation and regeneration of skeletal muscle: functional relevance of connexin43. J. Cell Sci. **118**, 27-37. (10.1242/jcs.01553) 15601660

[47] Merrifield PA, Laird DW. 2016 Connexins in skeletal muscle development and disease. Semin. Cell Dev. Biol. **50**, 67-73. (10.1016/J.SEMCDB.2015.12.001) 26688333

[48] Ricotti L et al. 2012 Proliferation and skeletal myotube formation capability of C2C12 and H9c2 cells on isotropic and anisotropic electrospun nanofibrous PHB scaffolds. Biomed. Mater. **7**, 035010. (10.1088/1748-6041/7/3/035010) 22477772

[49] Martins-Marques T, Catarino S, Zuzarte M, Marques C, Matafome P, Pereira P, Girão H. 2015 Ischaemia-induced autophagy leads to degradation of gap junction protein connexin43 in cardiomyocytes. Biochem. J. **467**, 231-245. (10.1042/BJ20141370) 25605500

[50] Hulikova A, Swietach P. 2016 Nuclear proton dynamics and interactions with calcium signaling. J. Mol. Cell. Cardiol. **96**, 26-37. (10.1016/j.yjmcc.2015.07.003) 26183898 PMC4915819

[51] De Smet MAJ et al. 2021 Cx43 hemichannel microdomain signaling at the intercalated disc enhances cardiac excitability. J. Clin. Invest. **131**, e137752. (10.1172/jci137752) 33621213 PMC8011902

[52] Lissoni A et al. 2021 RyR2 regulates Cx43 hemichannel intracellular Ca^2+^-dependent activation in cardiomyocytes. Cardiovasc. Res. **117**, 123-136. (10.1093/cvr/cvz340) 31841141

[53] Lissoni A, Wang N, Nezlobinskii T, De Smet M, Panfilov AV, Vandersickel N, Leybaert L, Witschas K. 2020 Gap19, a Cx43 hemichannel inhibitor, acts as a gating modifier that decreases main state opening while increasing substate gating. Int. J. Mol. Sci. **21**, 7340. (10.3390/IJMS21197340) 33027889 PMC7583728

[54] Ljubojevic S et al. 2014 Early remodeling of perinuclear Ca^2+^ stores and nucleoplasmic Ca^2+^ signaling during the development of hypertrophy and heart failure. Circulation **130**, 244-255. (10.1161/CIRCULATIONAHA.114.008927) 24928680 PMC4101040

[55] Esseltine JL, Laird DW. 2016 Next-generation connexin and pannexin cell biology. Trends Cell Biol. **26**, 944-955. (10.1016/j.tcb.2016.06.003) 27339936

[56] Hsu SC, Hung MC. 2007 Characterization of a novel tripartite nuclear localization sequence in the EGFR family. J. Biol. Chem. **282**, 10 432-10 440. (10.1074/JBC.M610014200) 17283074

[57] Luo H et al. 2023 Combinations of ivermectin with proteasome inhibitors induce synergistic lethality in multiple myeloma. Cancer Lett. **565**, 216218. (10.1016/J.CANLET.2023.216218) 37149018

[58] Turgay Y, Ungricht R, Rothballer A, Kiss A, Csucs G, Horvath P, Kutay U. 2010 A classical NLS and the SUN domain contribute to the targeting of SUN2 to the inner nuclear membrane. EMBO J. **29**, 2262-2275. (10.1038/emboj.2010.119) 20551905 PMC2910269

[59] Majoul I V, Onichtchouk D, Butkevich E, Wenzel D, Chailakhyan LM, Duden R. 2009 Limiting transport steps and novel interactions of Connexin-43 along the secretory pathway. Histochem. Cell Biol. **132**, 263-280. (10.1007/s00418-009-0617-x) 19626334 PMC2756399

[60] Solan JL, Lampe PD. 2014 Specific Cx43 phosphorylation events regulate gap junction turnover *in vivo*. FEBS Lett. **588**, 1423-1429. (10.1016/j.febslet.2014.01.049) 24508467 PMC3989505

[61] Fischer A, Spray DC, Brown AMC, Fishman GI, Ai Z. 2008 Wnt-1 regulation of connexin43 in cardiac myocytes. J. Clin. Investig. **105**, 161-171. (10.1172/jci7798) PMC377428 10642594

[62] Dukic AR, Gerbaud P, Guibourdenche J, Thiede B, Taskén K, Pidoux G. 2017 Ezrin-anchored PKA phosphorylates serine 369 and 373 on connexin 43 to enhance gap junction assembly, communication, and cell fusion. Biochem. J. **475**, 455-476. (10.1042/BCJ20170529) 29259079

[63] Tanaka S, Terada K, Nohno T. 2011 Canonical Wnt signaling is involved in switching from cell proliferation to myogenic differentiation of mouse myoblast cells. J. Mol. Signal. **6**, 12. (10.1186/1750-2187-6-12) 21970630 PMC3198762

[64] Cohen D et al. 2011 CXCL12 secretion by bone marrow stromal cells is dependent on cell contact and mediated by connexin-43 and connexin-45 gap junctions. Nat. Immunol. **12**, 391-398. (10.1038/ni.2017) 21441933

[65] Branco AF, Pereira SP, Gonzalez S, Gusev O, Rizvanov AA, Oliveira PJ. 2015 Gene expression profiling of H9c2 myoblast differentiation towards a cardiac-like phenotype. PLoS ONE **10**, e0129303. (10.1371/JOURNAL.PONE.0129303) 26121149 PMC4485408

[66] Delmar M, Laird DW, Naus CC, Nielsen MS, Verselis VK, White TW. 2018 Connexins and disease. Cold Spring Harb. Perspect. Biol. **10**, a029348. (10.1101/CSHPERSPECT.A029348) 28778872 PMC6120696

[67] Catarino S, Ramalho JS, Marques C, Pereira P, Girão H. 2011 Ubiquitin-mediated internalization of connexin43 is independent of the canonical endocytic tyrosine-sorting signal. Biochem. J. **437**, 255-267. (10.1042/BJ20102059) 21554242

[68] Almeida Paiva R et al. 2019 Ischaemia alters the effects of cardiomyocyte-derived extracellular vesicles on macrophage activation. J. Cell. Mol. Med. **23**, 1137-1151. (10.1111/jcmm.14014) 30516028 PMC6349194

[69] Ariotti N et al. 2015 Modular detection of GFP-labeled proteins for rapid screening by electron microscopy in cells and organisms. Dev. Cell **35**, 513-525. (10.1016/j.devcel.2015.10.016) 26585296

[70] Zuleger N, Kelly DA, Richardson AC, Kerr ARW, Goldberg MW, Goryachev AB, Schirmer EC. 2011 System analysis shows distinct mechanisms and common principles of nuclear envelope protein dynamics. J. Cell Biol. **193**, 109-123. (10.1083/jcb.201009068) 21444689 PMC3082195

[71] Landthaler M et al. 2008 Molecular characterization of human argonaute-containing ribonucleoprotein complexes and their bound target mRNAs. RNA **14**, 2580-2596. (10.1261/rna.1351608) 18978028 PMC2590962

[72] Joseph J, Tan SH, Karpova TS, McNally JG, Dasso M. 2002 SUMO-1 targets RanGAP1 to kinetochores and mitotic spindles. J. Cell Biol. **156**, 595-602. (10.1083/jcb.200110109) 11854305 PMC2174074

[73] Ciciarello M, Mangiacasale R, Thibier C, Guarguaglini G, Marchetti E, Di Fiore B, Lavia P. 2004 Importin *β* is transported to spindle poles during mitosis and regulates Ran-dependent spindle assembly factors in mammalian cells. J. Cell Sci. **117**, 6511-6522. (10.1242/jcs.01569) 15572412

[74] Howarth M, Liu W, Puthenveetil S, Zheng Y, Marshall LF, Schmidt MM, Wittrup KD, Bawendi MG, Ting AY. 2008 Monovalent, reduced-size quantum dots for imaging receptors on living cells. Nat. Methods **5**, 397-399. (10.1038/NMETH.1206) 18425138 PMC2637151

[75] Lam SS, Martell JD, Kamer KJ, Deerinck TJ, Ellisman MH, Mootha VK, Ting AY. 2015 Directed evolution of APEX2 for electron microscopy and proximity labeling. Nat. Methods **12**, 51-54. (10.1038/NMETH.3179) 25419960 PMC4296904

[76] Wilkie GS, Schirmer EC. 2008 Purification of nuclei and preparation of nuclear envelopes from skeletal muscle. Methods Mol. Biol. **463**, 23-41. (10.1007/978-1-59745-406-3_2) 18951158

[77] Chen Y, Sánchez A, Rubio ME, Kohl T, Pardo LA, Stühmer W. 2011 Functional kv10.1 channels localize to the inner nuclear membrane. PLoS ONE **6**, e19257. (10.1371/journal.pone.0019257) 21559285 PMC3086910

[78] Andronov L, Genthial R, Hentsch D, Klaholz BP. 2021 A spectral demixing method for high-precision multi-color localization microscopy. bioRxiv **5**, 473862. (10.1101/2021.12.23.473862) PMC9576791 36253454

[79] Andronov L, Lutz Y, Vonesch JL, Klaholz BP. 2016 SharpViSu: integrated analysis and segmentation of super-resolution microscopy data. Bioinformatics **32**, 2239-2241. (10.1093/BIOINFORMATICS/BTW123) 27153691 PMC4937188

[80] Kuleshov MV et al. 2016 Enrichr: a comprehensive gene set enrichment analysis web server 2016 update. Nucleic Acids Res. **44**, W90-W97. (10.1093/nar/gkw377) 27141961 PMC4987924

[81] Mak DOD, Vais H, Cheung KH, Foskett JK. 2013 Isolating nuclei from cultured cells for patch-clamp electrophysiology of intracellular Ca^2+^ channels. Cold Spring Harb. Protoc. **2013**, 880-884. (10.1101/PDB.PROT073056) 24003193 PMC3979423

[82] Martins-Marques T et al. 2023 Cx43 can form functional channels at the nuclear envelope and modulate gene expression in cardiac cells. Figshare. (10.6084/m9.figshare.c.6845650) PMC10645070 37907090

